# Identification of Marker Peptides for the Whey Protein Quantification in Edam-Type Cheese

**DOI:** 10.3390/foods12102002

**Published:** 2023-05-15

**Authors:** Tobias von Oesen, Mascha Treblin, Ingrid Clawin-Rädecker, Dierk Martin, Ronald Maul, Wolfgang Hoffmann, Katrin Schrader, Benjamin Wegner, Katja Bode, Ralf Zink, Sascha Rohn, Jan Fritsche

**Affiliations:** 1 Department of Safety and Quality of Milk and Fish Products, Max Rubner-Institut, Federal Research Institute of Nutrition and Food, Hermann-Weigmann-Straße 1, 24103 Kiel, Germany; tobias.vonoesen@mri.bund.de (T.v.O.); ingrid.clawin-raedecker@mri.bund.de (I.C.-R.); dierk.martin@mri.bund.de (D.M.); ronald.maul@mri.bund.de (R.M.); wolfgang.hoffmann@mri.bund.de (W.H.); katrin.schrader@mri.bund.de (K.S.); 2 Institute of Food Chemistry, Hamburg School of Food Science, University of Hamburg, Grindelallee 117, 20146 Hamburg, Germany; mascha.treblin@uni-hamburg.de (M.T.); rohn@tu-berlin.de (S.R.); 3 SGS Germany GmbH, Weidenbaumsweg 137, 21035 Hamburg, Germany; benjamin.wegner@sgs.com; 4 Center of Expertise Research & Technology (CoE-R&T), DMK Group (Deutsches Milchkontor GmbH), Flughafenallee 17, 28199 Bremen, Germany; katja.bode@dmk.de (K.B.); ralf.zink@dmk.de (R.Z.); 5 Department of Food Chemistry and Analysis, Institute of Food Technology and Food Chemistry, Technische Universität Berlin, TIB 4/3 1, Gustav Meyer Allee 25, 13355 Berlin, Germany

**Keywords:** dairy products, proteomic approach, food authenticity, food analysis, marker peptide selection, whey protein quantification

## Abstract

Several technologies are available for incorporating whey proteins into a cheese matrix. However, there is no valid analytical method available to determine the whey protein content in matured cheese, to date. Consequently, the aim of the present study was to develop a liquid chromatography-tandem mass spectrometry (LC-MS/MS) method for the quantification of individual whey proteins based on specific marker peptides (‘bottom-up’ proteomic approach). Therefore, the whey protein-enriched model of the Edam-type cheese was produced in a pilot plant and on an industrial scale. Tryptic hydrolysis experiments were performed to evaluate the suitability of identified potential marker peptides (PMPs) for α-lactalbumin (α-LA) and β-lactoglobulin (β-LG). Based on the findings, α-LA and β-LG appeared to be resistant to proteolytic degradation during six weeks of ripening and no influence on the PMP was observed. Good levels of linearity (R^2^ > 0.9714), repeatability (CVs < 5%), and recovery rate (80% to 120%) were determined for most PMPs. However, absolute quantification with external peptide and protein standards revealed differences in model cheese depending on the PMP, e.g., 0.50% ± 0.02% to 5.31% ± 0.25% for β-LG. As protein spiking prior to hydrolysis revealed differing digestion behavior of whey proteins, further studies are required to enable valid quantification in various cheese types.

## 1. Introduction

In 2020, global cheese production grew by 1.4% [1]. Cheese consumption varies between countries, but has increased considerably since 1970 [2]. Moreover, the increasing appreciation for and importance of whey proteins in various fields of application is leading to product innovations in the food industry, particularly in the dairy sector [3,4]. Instant whey powder or whey-based beverages are well-known and highly appreciated by consumers. However, semi-hard cheese is traditionally not known for its high whey protein content when produced according to the traditional production process. Nevertheless, the whey protein content of semi-hard cheese can be significantly increased in various ways, e.g., via heating up the initial milk (‘cheese milk’), membrane-based technologies, high hydrostatic pressure, ultrahigh-pressure homogenization, transglutaminase treatment, or hybrid variants of the aforementioned [5]. This enables consumers to obtain access to whey proteins without the necessity of consuming a completely new product. Already, the addition of microparticulated whey proteins to cheese milk was found to improve the texture of reduced-fat cheeses [6].

However, according to German food legislation, the fortification of traditional cheese with whey proteins is not yet permitted. Regarding possible future changes in food legislation, the availability of whey-protein-fortified dairy products requires valid analytical methods to ensure reliable product quality control and legal compliance. German food legislation is a pioneer in this respect. EU legislation or internationally recognized standards (e.g., IDF, Codex Alimentarius) might adapt their legislation or recommendations accordingly.

Protein analyses can be performed using a variety of techniques, such as immunology methods, electrophoresis, or chromatography, often coupled with mass spectrometric detection. Enzyme-linked immunosorbent assays (ELISA) use specific antibodies that react with the epitopes of antigens present in a target protein. Due to the commonly applied thermal treatment of milk over a wide temperature range during dairy processing, proteins may denature and subsequently lose their three-dimensional structural conformation. Epitopes can be altered, no longer being accessible for specific antibody reactions; underestimation could occur [7].

As there is no whey protein-free semihard cheese, a quantitative methodology for the determination of the whey protein content is required. Electrophoresis, especially capillary electrophoresis, is a well-established methodology for the quantification of casein in different matrices (e.g., milk of various mammals) [8]. However, cheese represents a complex matrix consisting of native ingredients and their (subsequent) degradation products, which limits electrophoresis in terms of sensitivity and selectivity [7]. Various liquid chromatography methods are commonly used to evaluate the heat treatment and to analyze the milk protein composition of milk and some other dairy products [9,10,11,12]. In addition, ripening-induced degradation products also prevented valid quantification of the whey protein content in ripened cheese when using liquid chromatography coupled with fluorescence detection [13].

Mass spectrometry-based proteomics became the most comprehensive approach for the quantitative profiling of proteins in recent years [14]. Methodological approaches in the field of proteomics are based on either ‘top-down’ or ‘bottom-up’ strategies. Top-down proteomics was already used for the identification and quantification of native major proteins in milk [15]. However, the identification or quantification of native milk proteins requires high-quality equipment and has not been successfully applied to a complex cheese matrix yet. In contrast, bottom-up (‘shotgun’) proteomics is characterized by an initial proteolytic in-vitro digestion of the native proteins prior to liquid chromatography-tandem mass spectrometry (LC-MS/MS) analysis. Due to its high catalytic activity and the formation of specific peptides with a basic arginine or lysine at the C-termini, trypsin is a widely used proteinase for enzymatic digestion [16]. The generated peptides may be unique for individual proteins and can be therefore used for the identification or quantification of the respective proteins. The bottom-up approach is regarded as a robust and high-throughput methodology that enables protein identification and quantification in complex hydrolysates [17].

In the field of LC-MS/MS analysis, the multiple reaction monitoring (MRM) data acquisition strategy further increased the quantification accuracy and reproducibility [18]. Recently, some MRM-LC-MS/MS methods were developed regarding milk proteomics. Specific marker peptides for individual milk proteins were used for species identification [19,20] and allergen detection [21,22]. Furthermore, studies regarding the quantification of individual milk proteins in milk [23], milk powder [24], infant formulas [25,26], or fresh cheese [27] were published. Moreover, an initial bottom-up approach for the quantification of milk proteins in various milk products, including Emmental-type cheese, was presented [28]. However, some of these methods are based on the assumption of equimolar hydrolysis of the selected marker peptide(s) for absolute quantification of the target protein. Recent studies on in-vitro hydrolysis and the stability of various marker peptides in milk powder showed that the digestibility of proteins and the subsequent hydrolysis of marker peptides differed significantly and should be considered for valid quantification in differing matrices [29]. Moreover, none of the mentioned studies investigated the proteolytic stability of the whey proteins during the cheese ripening of semi-hard cheese.

Consequently, the aim of this study was to identify potential marker peptides (PMPs) and to develop an MRM-LC-MS/MS method for an appropriate whey protein quantification in ripened Edam-type cheese using a bottom-up proteomic approach. In addition to linearity and repeatability experiments, recovery experiments were also performed to validate the MRM-LS-MS/MS peptide analysis. Moreover, the proteolytic stability of α-lactalbumin (α-LA) and β-lactoglobulin (β-LG) during the cheese ripening was monitored based on selected PMPs. Furthermore, enrichment factors (EF) for α-LA and β-LG in whey protein-enriched cheese were calculated based on the PMPs, and compared to the EF estimated as described by von Oesen et al. [13]. The α-LA and β-LG contents in the cheese were quantified using a certified milk protein standard mixture (MPSM) for both, external and internal calibration. To verify the suitability of the selected marker peptides for absolute quantification, the degree of hydrolysis (digestion behavior) of the whey proteins during the tryptic in-vitro digestion of MPSM was determined.

## 2. Materials and Methods

### 2.1. Materials

#### 2.1.1. Cheese Samples

Cheese samples from two different sample sets were used. Sample set I (‘pilot scale’) consisted of cheese samples manufactured in the pilot plant at the dairy technical center of the Max-Rubner Institut (MRI), Kiel, Germany. A standard model Edam-type cheese was produced from the same raw milk as a reference, in addition to every whey-protein-enriched cheese. The procedure for sample set I was already described by von Oesen et al. [13] and Treblin et al. [30]. Sample set II (‘industrial scale’) consisted of cheese samples manufactured at the pilot plant (Milk-Innovation Center (MIC) at Edewecht, Germany) of DMK Group Deutsches Milchkontor GmbH, Bremen, Germany, and was described by Treblin et al. [30]. An overview of the cheese samples analyzed in the present study is provided in Table 1.

#### 2.1.2. Protein and Peptide Standards

The following certified bovine protein standards (all purchased from Sigma–Aldrich Corp., St. Louis, MO, USA) were used to prepare a milk protein standard mixture (MPSM): α lactalbumin (α-LA, purity ≥ 85%), lactoferrin (LF, purity ≥ 85%), bovine serum albumin (BSA, purity ≥ 98%), β lactoglobulin A + B (β-LG, purity ≥ 90%), immunoglobulin G (IgG, purity ≥ 95%), α_s_-casein (α_s_-CN, purity ≥ 70%), β-casein (β-CN, purity ≥ 98%), and κ-casein (κ-CN, purity ≥ 70%). Phosphate buffer (10 mM, pH 6.7, prepared from disodium hydrogen phosphate and sodium dihydrogen phosphate, c.f., Section 2.1.3) was used to dissolve the protein standards. The MPSM was aliquoted and stored at −25 °C. Table 2 shows the protein concentrations for the MPSM (prepared following Farrell Jr. et al. [31]). Twenty-three synthetic peptide standards were purchased from Synpeptide Co., Ltd. (Shanghai, China) according to their sequences (Table 3).

#### 2.1.3. Reagents

Trypsin (from bovine pancreas, ≥ 10,000 BAEE units/mg protein), Tris (hydroxymethyl) aminomethane (purity: p.a.), and dl dithiothreitol (DTT; purity ≥ 99%), were purchased from Sigma–Aldrich Corp. (St. Louis, MO, USA). Urea (purity ≥ 99.5%), hydrochloric acid (37%), trifluoroacetic acid (TFA; purity ≥ 99.8%), acetonitrile, methanol (both HPLC (high-performance liquid chromatography) grade), disodium hydrogen phosphate (purity ≥ 99.99%), and sodium dihydrogen phosphate (purity ≥ 99.99%) were obtained from Merck KGaA (Darmstadt, Germany). Iodoacetamide (IAA; proteomics grade), acetonitrile (LC-MS grade), and water (LC-MS grade) were purchased from VWR International GmbH (Darmstadt, Germany). Formic acid (FA; LC-MS grade) was obtained from Altmann Analytik GmbH & Co. KG (Munich, Germany). Ultrapure water (0.055 µS) was obtained using a laboratory water purification system (Sartorius Arium^®^ 611 VF, Sartorius Lab Instruments GmbH & Co. KG, Göttingen, Germany). Urea was dissolved in 0.1 mol L^−1^ Tris-HCl (pH 7.8) to prepare the digestion buffer (6 mol L^−1^), DTT was dissolved in 0.1 mol L^−1^ Tris-HCl (pH 7.8) to prepare the reducing buffer (0.2 mol L^−1^), and IAA was dissolved in 0.1 mol L^−1^ Tris-HCl to prepare the alkylation buffer (0.2 mol L^−1^). Trypsin solution (2 mg mL^−1^) was prepared by dissolving trypsin in 0.1 mol L^−1^ hydrochloric acid.

### 2.2. Methods

#### 2.2.1. Protein Extraction

Protein extraction was carried out according to von Oesen et al. [13]. In brief, 7.5 g of shredded cheese was homogenized in 30 mL of 0.1 mol L^−1^ phosphate buffer (pH 6.7) for 3 min at room temperature using a disperser (Ultra-Turrax^®^, T25 digital, IKA^®^-Werke GmbH & Co. KG, Staufen, Germany). The resulting cheese phosphate buffer suspension (CPBS) was used for in-solution protein digestion (c.f., Section 2.2.2).

#### 2.2.2. In-Solution Protein Digestion

The samples were hydrolyzed with trypsin according to the Thermo Scientific^TM^ Pierce^TM^ digestion protocol [32] with the following procedure: a volume of 40 µL CPBS or 80 µL MPSM was added to 160 µL of digestion buffer. After adding 20 µL of reducing buffer, the samples were incubated for 1 h at 40 °C in a thermoshaker (MKR 23, DITABIS AG, Pforzheim, Germany) with a shaking speed of 400 min^−1^. Then, under light protection, 40 µL of alkylation buffer was added and incubated under shaking conditions (400 min^−1^) for 1 h at room temperature. Afterward, 20 µL of reducing buffer was added, mixed, and incubated for 30 min at room temperature without shaking. For dilution, prior to hydrolysis, 680 µL of water was added to the cheese samples and 640 µL of water was added to the MPSM. Finally, hydrolysis was started by adding 20 µL of trypsin solution. The samples were incubated under shaking conditions (400 min^−1^) at 37 °C for 16 h. The digestion reaction was quenched using 50 µL of 2 mol L^−1^ acetic acid. Finally, 500 µL of the protein hydrolysate was cleaned up via solid-phase extraction (SPE) using a Strata^TM^-X polymeric reversed phase (33 µm, 85 Å, 200 mg 6 mL^−1^; Phenomenex Ltd., Aschaffenburg, Germany). Conditioning of the cartridges was performed using methanol. Equilibration and washing were carried out with 0.1% (*v*/*v*) TFA in water, and elution was achieved by adding acetonitrile, water, and TFA at a ratio of 70:30:0.1 (*v*/*v*/*v*). The eluate was concentrated to dryness using a vacuum concentrator (Concentrator plus, Eppendorf SE, Hamburg, Germany), re-dissolved in 500 µL of a mixture of water, acetonitrile, and FA at a ratio of 98:2:0.2 (*v*/*v*/*v*), and finally aliquoted and stored at −80 °C. For LC-MS/MS analysis, 50 µL of the aliquoted fluid was diluted by a factor of 20 with the previously mentioned mixture of water, acetonitrile, and FA.

#### 2.2.3. Data-Dependent Acquisition Liquid Chromatography-Tandem Mass Spectrometry (DDA-LC-MS/MS)

The determination of the peptide profiles was performed with LC-MS, using an HPLC system (UltiMate^TM^ 3000 RSLCnano system, Thermo Fisher Scientific, Bremen, Germany) and an LTQ XL^TM^ linear ion trap mass spectrometer (Thermo Fisher Scientific GmbH, Bremen, Germany). For chromatographic separation, a PepSwift^TM^ (Thermo Fisher Scientific, Bremen, Germany ) monolithic capillary LC column (200 µm × 25 cm nanoViper^TM^ column; Thermo Fisher Scientific, Bremen, Germany) with a PepSwift^TM^ monolithic guard column (200 µm × 5 mm nanoViper^TM^ column; Thermo Fisher Scientific, Bremen, Germany) was used at a flow rate of 1 µL min^−1^ and a column temperature of 40 °C. Gradient elution was carried out with a mixture of two solvents. Solvent A consisted of water with 0.1% (*v*/*v*) FA and solvent B was made of water and acetonitrile at a ratio of 20:80 (*v*/*v*) with 0.1% (*v*/*v*) FA. The solvent gradient program started with isocratic elution at 1% solvent B for 1 min, followed by a linear gradient by increasing the proportion of solvent B at 0.83% min^−1^ (59 min) and 20% min^−1^ (2 min). Then, isocratic elution at 90% solvent B took place for 5 min and returned to the initial conditions within 0.2 min for a 12.8 min re-equilibration. The samples were kept at 5 °C until injection. The injection volume was 1 µL. The MS experiments were performed in the positive ion mode using nanospray ionization with a spray voltage of 2 kV, tube lens voltage of 110 V, and a capillary temperature of 200 °C. A data-dependent scan with a full-scan range from 300 to 2000 *m*/*z* and fragmentation of the three most intense ions (activation type = CID (35 eV)) with an isolation width of 2 Da was performed. The activation Q was 0.25.

#### 2.2.4. In-Silico Peptide Identification

MS data resulting from a data-dependent LC-MS/MS analysis (c.f., Section 2.2.3) of cheese samples after 6 weeks of ripening from sample set I, containing 30% high-heat milk (HH milk), were used for peptide identification. MS data interpretation was performed with the Proteome Discoverer^TM^ 1.4 (Thermo Fisher Scientific GmbH, Bremen, Germany), using the search algorithm SEQUEST-HT with the following search parameter settings: enzyme name: trypsin (full); max. missed cleavage site: two; minimum peptide length: four; maximum peptide length: 144; precursor mass tolerance: 1.5 Da; fragment mass tolerance: 0.8 Da; dynamic modifications: oxidation (M), phosphorylation (S, T, Y), dihexoses (K, R); static modifications: carbamidomethyl (C). The database of UniProtKB from February 2017 (UniProt KB), restricted to Bos Taurus, was used to identify peptides according to their MS values. The identified peptides were manually verified (c.f., Section 2.2.5).

#### 2.2.5. Peptide Selection

Peptides released from the major whey proteins present in milk, α-LA, and β-LG were considered. The identified peptide sequences were manually verified by comparing the measured fragment spectra of the peptides with the theoretical peptide fragments. The criteria for the peptide selection were as follows: the spectra had to match at least five y- or b-ions of the theoretical peptide fragments (‘fragment matches’). It was considered that modifications, such as phosphorylation, water, or ammonium adducts, were allowed to be present only to a small extent. Furthermore, all major fragment masses of the spectra with intensities greater than 10–20% of the maximum intensity in the MS/MS had to match theoretical peptide fragments. Additionally, the number of peptide spectrum matches had to be at least two for each identified peptide. Moreover, the molecular weight was expected not to exceed 2500 Da and the number of amino acids was expected to be at least 4.

#### 2.2.6. Multiple Reaction Monitoring Liquid Chromatography-Tandem Mass Spectrometry (MRM-LC-MS/MS)

The three fragments with the greatest intensity in the MS^2^ spectra of each peptide were selected from the result table provided by Proteome Discoverer^TM^. Their *m*/*z* values were implemented via an MRM-LC-MS/MS method. The sequence of the PMPs as well as their corresponding retention time and the specific *m*/*z* value for the precursor and the fragments are listed in Table 3. The peptides were separated on an HPLC system (Infinity series, Agilent Technologies Deutschland GmbH, Waldbronn, Germany) equipped with a Hypersil GOLD^TM^ aQ C18 column (3 µm, 150 mm × 2.1 mm) and guard column (Hypersil GOLD^TM^ C18 column, 5 µm, 10 mm × 2.1 mm; both columns: Thermo Fisher Scientific GmbH, Bremen, Germany) with a flow rate of 0.3 mL min^−1^ for 40 min, and a column temperature of 40 °C. The samples were kept at 4 °C. Gradient elution was carried out with a mixture of two solvents. Solvent A consisted of water with 0.1% (*v*/*v*) FA and solvent B was made of acetonitrile with 0.1% (*v*/*v*) FA. The solvent gradient program started with an isocratic elution at 2% solvent B for 3 min, followed by a linear gradient induced by increasing the proportion of solvent B at 2.7% min^−1^ (3 min), 0.83% min^−1^ (12 min), 1.42% min^−1^ (12 min), and 265% min^−1^ (0.2 min). Then, isocratic elution at 90% solvent B took place for 2.4 min and returned to the initial conditions within 0.2 min for a 7.2-min re-equilibration. The Infinity HPLC system was coupled with a Sciex QTrap^®^ 6500+ mass spectrometer (Sciex Germany GmbH, Darmstadt, Germany) using an electrospray ionization (ESI) interface. The HPLC eluent of the first 6 min and of the last 12 min were diverted to waste. The TurboIonSpray^®^ conditions were as follows: curtain gas^TM^: 40 psi, collision gas: medium, ion spray voltage: 5000 V, temperature: 300 °C, ion source gas 1 was 65 psi, and ion source gas 2 was 70 psi. The mass spectrometer was operated in a multiple reaction monitoring (MRM) quadrupole mode scan type with high mass configuration (5–2000 Da) in a positive ion mode.

#### 2.2.7. Evaluation of the MRM-LC-MS/MS Method

The linearity, repeatability, and recovery rate of the PMPs were determined. For the determination of linearity, a synthetic peptide standard mixture was prepared comprising 23 synthetic peptides (c.f., Section 2.1.2, and Table 3), and was subsequently used for MRM-LC-MS/MS analysis at 3 concentration levels ranging from 54.4 ng mL^−1^ to 108.8 ng mL^−1^. For the evaluation of linearity, the coefficient of determination (R^2^) was calculated.

In addition, 8-fold (cheese) and 5-fold (MPSM) tryptic hydrolyses were performed to determine the repeatability of the tryptic hydrolyses of differing matrices. In addition, one tryptic hydrolysate of both cheese and MPSM was measured ten times to determine the repeatability of the MRM-LC-MS/MS analysis. Moreover, the synthetic peptide standard mixture was injected ten times to determine the repeatability of MRM-LC-MS/MS analysis. The coefficient of variation (CV) was calculated to evaluate repeatability.

Both hydrolysates of 6 weeks ripened cheese from sample set I containing 30% HH milk and hydrolysates of MPSM were spiked with the synthetic peptide standard mixture (c = 4.35 µg mL^−1^) at three spiking levels (c = 54.4, 81.8, 108.8 ng mL^−1^). Sample spiking was carried out as shown in Table S1, followed by solid-phase extraction (c.f., Section 2.2.2) and MRM-LC-MS/MS analysis (c.f., Section 2.2.6). In addition, the synthetic peptide mixture was mixed with water instead of matrix hydrolysates (Table S2). These matrix-free samples were used for MRM-LC-MS/MS analysis without SPE before. The recovery rate of the PMP upon spiking the hydrolysates of CPBS and MPSM (%RC_peptide spiked hydrolysate_) with a synthetic peptide standard mixture was calculated as the mean of all spiking levels according to Equation (1). Here, A_PMP spiked hydrolysate_ is the area of the peptide-spiked hydrolysate of CPBS or MPSM in levels 1, 2, or 3; A_PMP non-spiked hydrolysate_ stands for the area of the non-spiked hydrolysate of CPBS or MPSM; and A_PMP peptide mix_ represents the area of the synthetic peptide standard mixture without SPE at levels 1, 2, or 3.

(1) $${\%\mathrm{RC}}_{\mathrm{peptide}\;\mathrm{spiked}\;\mathrm{hydrolysate}}=\frac{\left(A_{\mathrm{PMP}\;\mathrm{spiked}\;\mathrm{hydrolysate}}-{\;A}_{\mathrm{PMP}\;\mathrm{non}-\mathrm{spiked}\;\mathrm{hydrolysate}}\right)\;\times \;100\%}{A_{\mathrm{PMP}\;\mathrm{peptide}\;\mathrm{mix}}}$$

#### 2.2.8. Calculation of Enrichment Factors

Cheeses from sample sets I and II were tryptically hydrolyzed after six weeks of ripening and they were analyzed via MRM-LC-MS/MS. The obtained peak areas were plotted against the amount of HH milk, and a linear regression as well as a coefficient of determination (R^2^) was determined for each PMP. The ratio of the peak area of the PMPs in cheese containing 10%, 20%, or 30% HH milk in relation to the peak area of the PMPs in standard cheese (0% HH milk) was termed ‘enrichment factor’ (EF) and was calculated according to Equation (2). Here, ‘b’ is the slope of the regression line, ‘x’ represents the amount of HH milk in cheese milk, and ‘a’ is the *y*-axis intercept at 0% HH milk.

(2) $$\mathrm{EF}=\frac{b\;\times \;x+a}{a}$$

#### 2.2.9. Determination of α-Lactalbumin and β-Lactoglobulin Content in Cheese via External Calibration

A milk protein standard mixture (MPSM; c.f., Section 2.1.2) was used as an external protein standard for the quantification of α-LA and β-LG in cheese. Therefore, MPSM and cheese phosphate buffer suspension (CPBS) were hydrolyzed by trypsin, cleaned up, and prepared for MRM-LC-MS/MS analysis, as described in Section 2.2.2 and Section 2.2.6. Finally, the individual whey protein content (α-LA and β-LG) in the cheese (%IWPC_cheese_) was calculated according to Equation (3). Here, A_PMP cheese_ and A_PMP MPSM_ are the areas of the whey-protein-specific PMPs in cheese and MPSM, respectively; c_IWPC MPSM_ stands for the concentration of the respective whey protein in the MPSM; m_cheese CPBS_ is the mass of the weighed cheese present in the volume of the CPBS (V_CPBS_) (c.f., Section 2.2.1); and DF_hydrolysis cheese_ and DF_hydrolysis MPSM_ represent the dilution factor during the hydrolysis of cheese and MPSM, respectively. The dilution factors for the hydrolyses of cheese and MPSM were 25.75 and 12.88, respectively.

(3) $${\%\mathrm{IWPC}}_{\mathrm{cheese}}=\frac{A_{\mathrm{PMP}\;\mathrm{cheese}}{\;\times \;c}_{\mathrm{IWPC}\;\mathrm{MPSM}}{\;\times \;\mathrm{DF}}_{\mathrm{hydrolysis}\;\mathrm{cheese}}{\;\times \;V}_{\mathrm{CPBS}}\;\times \;100\%}{A_{\mathrm{PMP}\;\mathrm{MPSM}\;}{\times \;\mathrm{DF}}_{\mathrm{hydrolysis}\;\mathrm{MPSM}}{\;\times \;m}_{\mathrm{cheese}\;\mathrm{CPBS}}}$$

#### 2.2.10. Degree of Hydrolysis of Potential Marker Peptides during Tryptic In-Vitro Hydrolysis of Milk Protein Standard Mixture

The digestibility was evaluated by calculating the degree of hydrolysis for the PMPs (%DH_PMP_) to be drawn out of the proteins α-LA and β-LG from the MPSM, according to Equation (4). Here, n_PMP MPSM_ is the amount of substance (mole) of the PMPs determined to be in the hydrolysate of the MPSM using a synthetic peptide standard mixture; n_MPSM_ represents the amount of substance (mole) of the respective protein present in the MPSM before hydrolysis.

(4) $${\%\mathrm{DH}}_{\mathrm{PMP}}=\frac{n_{\mathrm{PMP}\;\mathrm{MPSM}}\;\times \;100\%}{n_{\mathrm{MPSM}}}$$

#### 2.2.11. Determination of the Recovery Rate by Adding Milk Protein Standard Mixture to Cheese Phosphate Buffer Suspension

Cheese containing 30% HH milk after 6 weeks of ripening from sample set I was used to prepare a cheese phosphate buffer suspension (CPBS; c.f., Section 2.2.1). MPSM was used for the protein spiking of CPBS prior to tryptic hydrolysis (c.f., Table S3). Here, 2 mg of cheese was spiked with 28.9 µg of β-LG and 10.8 µg of α-LA (spiking level 1), 57.5 µg of β-LG and 21.5 µg of α-LA (spiking level 2), and 86.8 µg of β-LG and 32.3 µg of α-LA (spiking level 3). In addition to the protein-spiked samples, the CPBS and the MPSM were used separately for hydrolysis by trypsin. The volumes used for the hydrolyses are shown in Table S4. The volume of water mentioned in Table S4 refers to the dilution before the addition of trypsin, as described in Section 2.2.2. The hydrolyses were performed in duplicate. The subsequent MRM-LC-MS/MS analyses were carried out once. The preparation of the spiked samples resulted in a dilution of the CPBS (Table S3). Additionally, 40 µL of the spiked samples was used for hydrolysis, whereas only 20 µL of the CPBS, and 20, 40, and 60 µL of the MPSM were used for hydrolysis (Table S4). Consequently, these factors were considered before the recovery rate was calculated. The recovery rate of the PMP in CPBS upon protein spiking and before hydrolysis (%RC_protein spiked CPBS_) with MPSM was calculated as the mean of all spiking levels according to Equation (5). Here, A_PMP protein spiked_ is the area of the MPSM spiked sample in levels 1, 2, or 3; A_PMP cheese_ stands for the area of the non-spiked CPBS sample; and A_PMP MPSM_ represents the area of the MPSM in levels 1, 2, or 3.

(5) $${\%\mathrm{RC}}_{\mathrm{protein}\;\mathrm{spiked}\;\mathrm{CPBS}}=\;\frac{\left(A_{\mathrm{PMP}\;\mathrm{protein}\;\mathrm{spiked}\;\mathrm{CPBS}\;}-\;A_{\mathrm{PMP}\;\mathrm{CPBS}}\right)\;\times \;100\%}{A_{\mathrm{PMP}\;\mathrm{MPSM}}}$$

#### 2.2.12. Determination of α-Lactalbumin and β-Lactoglobulin Content in Cheese via Internal Calibration

Both the CPBS and the MPSM-spiked CPBS (level 1–3) mentioned in Section 2.2.11 and Tables S3 and S4 were used to determine the α-LA and β-LG content of cheese via a protein calibration in the cheese matrix. Therefore, the peak areas of the PMP were plotted against the volume of MPSM present in the sample volume used for tryptic hydrolysis (Table S4). Based on linear regression, the *y*-axis intercept ‘a_PMP_’ and the slope ‘b_PMP_’ were determined, and they were subsequently used for the calculation of the individual whey protein content (α-LA and β-LG) in the cheese (%IWPC_cheese_), according to Equation (6). Here, c_IWPC MPSM_ stands for the concentration of the respective whey protein in the MPSM (Table 2), m_cheese CPBS_ is the mass of the weighed cheese present in the volume of the CPBS (V_CPBS_) (c.f., Section 2.2.1), and V_CPBS spiking_ is the volume of CPBS present in the sample volume of the spiked samples (c.f., Table S4).

(6) $${\%\mathrm{IWPC}}_{\mathrm{cheese}}=\frac{a_{\mathrm{PMP}}{\;\times \;c}_{\mathrm{IWPC}\;\mathrm{MPSM}}{\;\times \;V}_{\mathrm{CPBS}}\;\times \;100\%}{b_{\mathrm{PMP}}{\;\times \;V}_{\mathrm{CPBS}\;\mathrm{spiking}}{\;\times \;m}_{\mathrm{cheese}\;\mathrm{CPBS}}}$$

#### 2.2.13. Two-Sample *t*-Test (Student’s *t*-Test)

A two-sample *t*-test (two-sided) was used to determine if a significant difference between the means of the two datasets was present (nondirectional hypothesis). Here, the hypotheses were as follows: the null hypothesis was that the means were equal; the alternate hypothesis was that the means were not equal. In addition, a two-sample *t*-test (one-sided) was used to determine if the mean of one dataset (dataset A) was greater or less than the mean of another dataset (dataset B) (directional hypothesis). Here, the hypotheses were as follows: the null hypothesis was that the mean of dataset A was less than or equal to the mean of dataset B; the alternate hypothesis was that the mean of dataset A was significantly greater than the mean of dataset B. A two-sample *t*-test was performed using Microsoft^®^ Excel^®^ for Microsoft 365 MSO (Version 2302, Microsoft Corporation, Redmond, WA, USA). The calculated t value was compared to the critical t value (t table, *p* < 0.05). The null hypothesis was rejected when the calculated t value was higher than the table value. Thus, the result was statistically significant (*p* < 0.05) [33].

## 3. Results and Discussion

### 3.1. Repeatability, Linearity, and Recovery Rate for the Determination of the Potential Marker Peptides

To validate the MRM-LC-MS/MS analytical method, a synthetic peptide standard mixture (c.f., Section 2.1.2), and an SPE-purified tryptic hydrolysate of both, six-week ripened cheese containing 30% HH milk and the MPSM, was measured ten times in a row to determine the repeatability of the MRM-LC-MS/MS analysis. The coefficient of variation (CV, %) was calculated for the peak area of the PMPs. Here, the CV for all peptides, except for the semi-tryptic peptide PMHIR, was below 10% for all three matrices. Most peptides even showed a repeatability of less than 2% (Table 4). An effect of the different matrices on the CV was not observed except for the peptide PMHIR and some hydrophobic and acidic peptides (peptides 19, 20, 22, and 23), which showed higher variations, especially in the low-concentrated aqueous standard solution.

Furthermore, the repeatability of the tryptic hydrolyses of both the cheese containing 30% HH milk and the MPSM was determined to consider the sample preparation, especially the enzymatic hydrolysis. Therefore, an eight-fold repeated tryptic hydrolysis of a cheese sample and a five-fold repeated tryptic hydrolysis of MPSM were performed. As expected, the repeatability of the tryptic hydrolysates increased slightly in comparison to the analytical variations. The CV of most PMPs was still low and ranged from approximately 1% to approximately 4%, indicating a good repeatability of both, the tryptic hydrolyses and the developed sample preparation protocol. Regarding the tryptic hydrolyses of the cheese sample, the peptide IPAVFK showed the lowest CV (0.78%). Only three PMPs showed CVs of more than 10%, and ranged from 11.08% (VGINYWLAHK; *m*/*z* 600.5/931.2) to 13.68% (VGINYWLAHK; *m*/*z* 400.7/551.3). Regarding the tryptic hydrolyses of MPSM, the peptide TPEVDDEALEKFDK showed the lowest CV (1.62%). Here, the peptide DDQNPHSSNIcNIScDK showed the highest CV (8.94%). For both matrices, the CV of the peptide PMHIR was not considered due to its semi-tryptic character.

Moreover, a synthetic peptide standard mixture was analyzed at three concentration levels, ranging from 54.9 ng mL^−1^ to 109 ng mL^−1^, to verify the linear measurement range. The coefficient of determination (R^2^) of the linear regression of the PMP ranged from 0.9714 (TPEVDDEALEKFDK) to 1 (LSFNPTQLEEQcHI).

A synthetic peptide standard mixture was also used to spike both CPBS hydrolysates and MPSM hydrolysates to verify a possible matrix effect of the hydrolysates on the subsequent SPE purification and MRM-LC-MS/MS analysis. Prior to SPE purification, the synthetic peptide standard mixture was added to both, CPBS hydrolysate and MPSM hydrolysate at three concentration levels. Finally, the recovery rate was calculated. The recovery rate of most PMPs ranged from 70% to 130%, considering the standard deviation (SD). Therefore, these PMPs are chemically stable enough for subsequent analytical procedures after tryptic digestion [29]. However, at least one PMP for β-LG (peptide no. 14) and four peptides for α-LA (peptide no. 16, 19, and 20) are out of this range (Table 5).

The recovery rate of these peptides was obviously affected by the matrix, e.g., during the SPE purification and/or during the mass spectrometric detection. For recovery rates significantly lower than 100%, the matrix effect may influence the SPE purification, but also a reduced ionization in the complex hydrolysate matrix is possible. For recovery rates higher than 100%, it can be assumed that the peptides were stabilized and more effectively ionized in the matrix of the CPBS or MPSM hydrolysates than in the low-concentrated aqueous synthetic peptide standard mixture. Three long hydrophobic peptides (peptides no. 12, 22, and 23) showed large differences in the peak areas between the aqueous synthetic peptide standard mixture and the CPBS and MPSM hydrolysates. This did not allow for a meaningful calculation of the recovery rates in the applied concentration levels of the spiked samples. Although a good recovery rate is not necessary for a valid quantification, the matrix effects must be considered and may lead to problems with quantification in different product matrices.

Nevertheless, the MRM-LC-MS/MS method applied showed reasonable recovery rates, coefficients of determination, and coefficients of variation for most PMPs.

### 3.2. Stability of Potential Marker Peptides during Cheese Ripening

**Figure 1:**
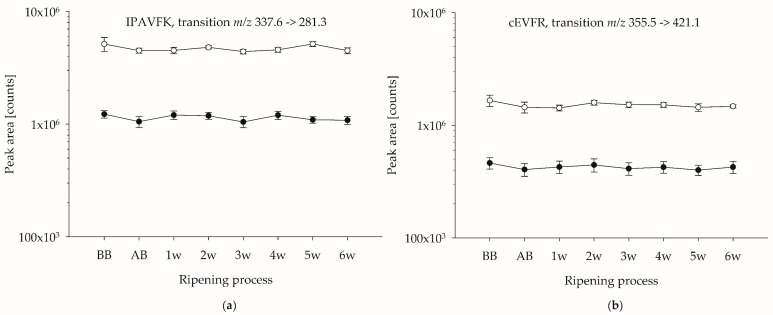
Peak areas of PMPs throughout the ripening process of cheese from sample set I. (**a**) PMP for β-LG; (**b**) PMP for α-LA. For graphical representation, the *y*-axis was displayed logarithmically. (●) Cheese containing 0% HH milk; (○) cheese containing 30% HH milk. Tryptic hydrolyses were performed in duplicate and measured once.

A prerequisite for suitable marker peptides deemed appropriate for the quantification of the whey protein content in ripened cheese is their proteolytic stability during cheese ripening. To the best of our knowledge, there are no data in the literature on the ripening-induced degradation of both, β-LG and α-LA, and thus, on the stability of their specific marker peptides in Edam-type cheese. For the evaluation of the proteolytic stability, tryptic hydrolyses of a cheese containing 30% HH milk and its corresponding standard cheese were performed before brining (BB), after brining (AB), and weekly, until the sixth week of ripening (sample set I). For sample set II, tryptic hydrolyses of one cheese containing 30% HH milk and its corresponding standard cheese were performed BB, AB, and after three and six weeks of ripening. MRM-LC-MS/MS was used to determine the peak area of the PMPs. Figure 1 shows exemplarily the peak area throughout the ripening process of one PMP for β-LG and one PMP for α-LA in the cheese of sample set I. The peak areas of additionally monitored PMP during ripening are shown in Figure S1 (sample set I) and Figure S2 (sample set II). Although some variations in the peak areas of the PMPs throughout the ripening process were noticed, a decreasing trend was not observed, indicating reasonable proteolytic stability of the whey proteins β-LG and α-LA, and a constant hydrolysis of the PMPs in Edam-type cheese at any monitored ripening time. Throughout the monitored ripening process, the peak area of the PMPs for both β-LG and α-LA were increased for the cheese containing 30% HH milk compared to the standard cheese.

### 3.3. Enrichment Factors of Individual Whey Proteins

Both the standard cheese and the cheeses containing 10%, 20%, and 30% HH milk obtained from sample set I and sample set II, were tryptically hydrolyzed after six weeks of ripening and subsequently analyzed via MRM-LC-MS/MS (c.f., Section 2.2.8). The peak area of the PMPs was plotted against the HH milk content, and a linear regression was performed (Figure S3). All selected PMPs, with the exception of PMHIR, showed a linear increase with increasing HH milk content in both sample sets, indicating a higher whey protein content. In principle, all PMPs allowed a relative quantification of β-LG or α-LA in relation to the standard cheese sample. The coefficients of determination (R^2^) ranged between 0.9335 and 0.9999 (sample set I).

Enrichment factors (EF) for the individual whey proteins were calculated as mean values based on the linear regression of fourteen PMPs for β-LG and eight PMPs for α-LA, respectively (Table 6 and Table 7). The best correlation between the β-LG content and the PMP was found for the peptide TKIPAVFK (*m*/*z* 452.0/674.2; R^2^ = 0.9999). For α-LA, the best correlation was found for the peptide cEVFR (*m*/*z* 355.5/421.1; R^2^ = 0.9996) (Figure S3).

As expected, both proteins β-LG and α-LA were enriched in cheese containing 10%, 20%, and 30% HH milk compared to their states in the standard cheese. The EFs of both, β-LG and α-LA, increased with the amount of HH milk added to cheese milk. However, compared to the EF of β-LG, the EF of α-LA was significantly lower (*p* < 0.05) for all whey-protein-enriched cheeses containing HH milk. As β-LG is a more heat-sensitive protein than α-LA, more intense heat-induced denaturation, and subsequent enrichment of denatured β-LG in the cheese matrix occurred [9,13].

In addition, the EFs for α-LA and β-LG, determined based on the PMPs, were compared to the estimated EFs, calculated based on the degree of individual whey protein denaturation in the cheese milk [13]. No significant difference (*p* < 0.05) was found between the EFs for α-LA and β-LG, determined based on the PMPs, and the estimated EFs. In principle, all PMPs except PMHIR are suitable for the relative quantification of β-LG or α-LA in relation to the standard cheese, and they differ only in their precision. Consequently, the relative quantification of individual whey protein content using the identified PMPs is valid within the same cheese matrix.

### 3.4. Quantification of α-Lactalbumin and β-Lactoglobulin in Model Edam-Type Cheese

In recently published studies selected marker peptides have been used for quantifying β-LG and α-LA in various milk products, such as raw milk, milk powder, infant formulas, and fresh cheese [24,25,26,27,28,29]. For example, the marker peptide TPEVDDEALEK has been used to quantify β-LG in milk powder [24] and fresh cheese [27]. The peptides VGINYWLAHK, LDQWLcEK, and cEVFR have been used to quantify α-LA in raw milk [25] and infant milk formula [26]. Based on a bottom-up proteomic approach using isotopically labeled peptides as internal standards, Bär et al. [28] presented a multiple-reaction monitoring liquid chromatography-mass spectrometry (MRM-LC-MS/MS) method that enables the simultaneous quantification of twenty milk proteins in various dairy products, e.g., in Swiss Emmental (which is a raw milk cheese). There, the marker peptides ALPMHIR and VGINYLWLAHK were used to quantify β-LG and α-LA, respectively. However, to the best of our knowledge, the matrix effect of the complex cheese matrix and cheese ripening-induced degradation of both the whey proteins and the selected marker peptides were not analyzed.

**Figure 2:**
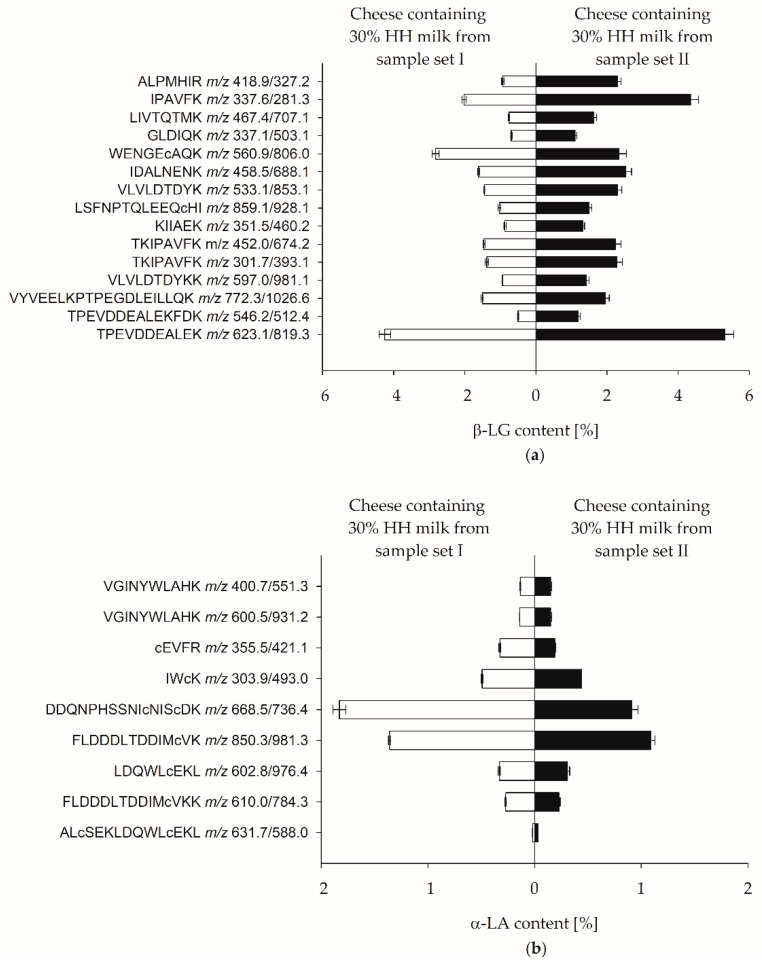
Quantification based on external protein calibration. (**a**) Mean ± SD for marker peptide-specific β-LG content of cheese containing 30% HH milk from sample set I and sample set II. Tryptic hydrolyses were performed in duplicate and measured once. (**b**) Mean ± SD for marker peptide-specific α-LA content of cheese containing 30% HH milk from sample set I and sample set II, tryptic hydrolyses in duplicate and measured once.

As these previous studies came partly to contradictory results for the selection of suitable marker peptides, the quantification of α-LA and β-LG in Edam-type cheese was initially carried out based on all identified PMPs in the present study (c.f., Table 3). Figure 2 shows the determined β-LG and α-LA content in cheese, based on the PMPs. Quantification was performed using MPSM for external calibration with a protein composition comparable to milk (c.f., Table 2).

Depending on the individual PMPs, the β-LG content ranged from 0.50% ± 0.018% (TPEVDDEALEKFDK) to 5.31% ± 0.25% (TPEVDDEALEK) and the α-LA content ranged from 0.02% ± 0.001% (ALcSEKLDQWLcEKL) to 1.83% ± 0.06% (DDQNPHSSNIcNIScDK). These results indicate that the accuracy of protein quantification using the identified PMPs was affected by matrix effects during SPE purification and/or ionization during the MRM-LC-MS/MS analysis of the cheese sample and the MPSM.

Consequently, valid whey protein quantification was not possible based on external calibration with MPSM. However, the β-LG and α-LA contents of the cheeses obtained from both sample sets showed comparable patterns for the different PMPs.

**Figure 3:**
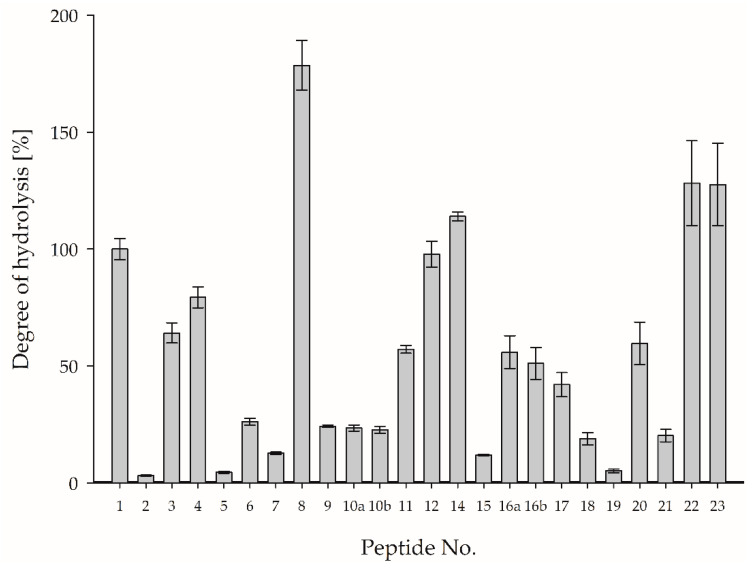
Mean ± SD for the degree of hydrolysis of PMPs during five tryptic in-vitro hydrolyses of MPSM. For corresponding peptide sequences, refer to Table 3.

Hao et al. [29] pointed out that a well-defined quantitative relationship between a protein and its corresponding marker peptide(s) after tryptic digestion is recommended for valid protein quantification. Consequently, the digestibility of the whey proteins as an important factor affecting the accuracy of protein quantification was assessed by calculating the degree of hydrolysis of the PMPs. Even for optimized hydrolysis conditions, not all tryptic cleavage sites of the whey proteins could be completely hydrolyzed and no equimolar relation to the marker peptide was observed. To verify the degree of hydrolysis, a MPSM with known amounts of proteins was used (c.f., Section 2.2.10). Figure 3 shows the degree of hydrolysis of the PMPs for α-LA and β-LG, based on the digestion of MPSM, indicating a large variation in the degree of hydrolysis. Most of the PMPs ranged from approximately 3% (IPAVFK) to approximately 100% (ALPMHIR). However, one PMP for β-LG and three PMPs for α-LA showed degrees of hydrolysis of more than 100%, also indicating matrix effects between the MPSM and the aqueous synthetic peptide standard mixture used for quantification.

**Figure 4:**
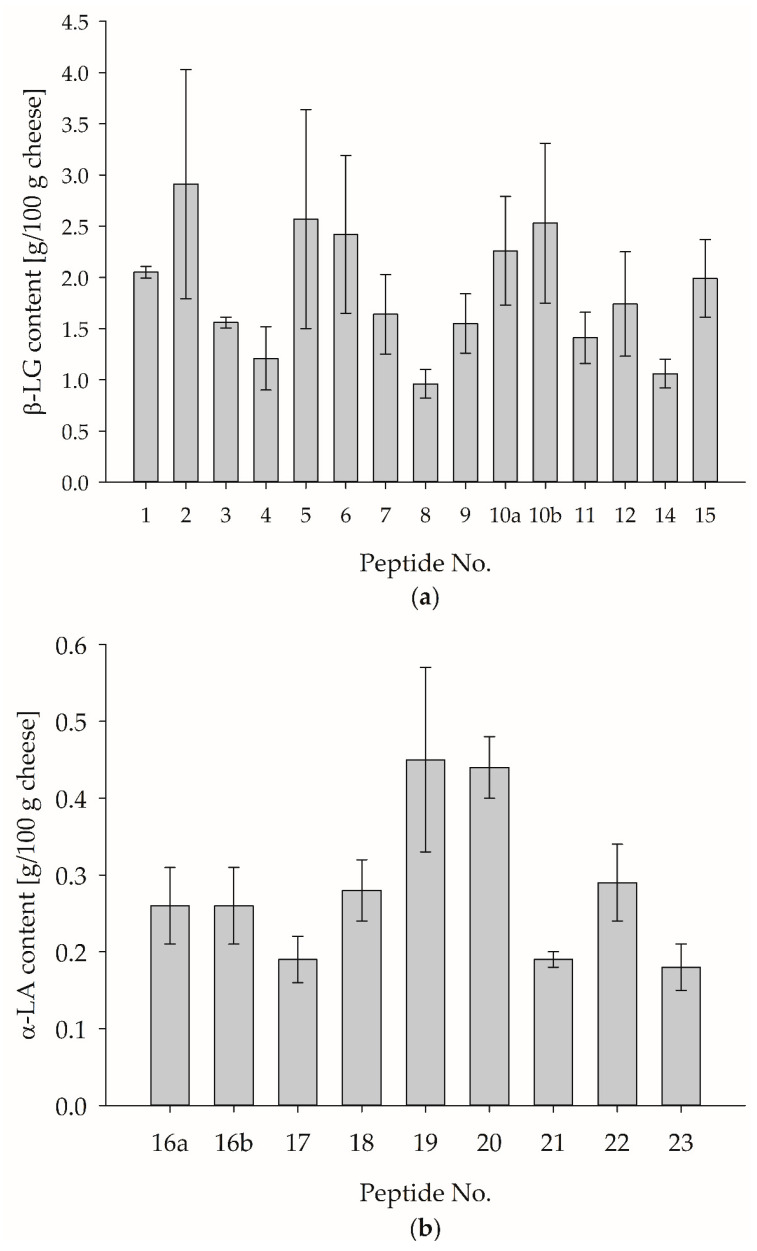
Quantification of β-LG and α-LA in cheese containing 30% HH milk from sample set I based on internal protein calibration in cheese matrix. Tryptic hydrolyses were performed in duplicate and measured once. (**a**) Mean ± SD for the marker peptide-specific β-LG content. (**b**) Mean ± SD for the marker peptide-specific α-LA content. For corresponding peptide sequences, refer to Table 3.

The results showed that an internal calibration of the cheese matrix (spiking with MPSM) prior to tryptic in-vitro hydrolyses is necessary for a valid whey protein quantification to guarantee that the PMPs in both matrices, MPSM, and cheese, were hydrolyzed in the same ratio and no matrix effect distorted the results. Therefore, both, the β-LG and α-LA content in cheese containing 30% HH milk from sample set I, were determined via internal protein calibration (c.f., Section 2.2.12). Figure 4a,b show the results for the marker peptide-specific β-LG and α-LA content.

Depending on the individual PMP, the β-LG content ranged from 0.96% ± 0.14% (LSFNPTQLEEQcHI) to 2.91% ± 1.12% (IPAVFK), and the α-LA content ranged from 0.18% ± 0.03% (ALcSEKLDQWLcEKL) to 0.45% ± 0.12% (DDQNPHSSNIcNIScDK). Compared to the external calibration, the determined β-LG and α-LA contents varied in a smaller range. However, the standard deviation (SD) for most PMPs is high, making it difficult to select a suitable PMP.

In a previous study, reference values for β-LG and α-LA contents in the analyzed cheese were already estimated [13]. Here, a β-LG content of 1.32% and an α-LA content of 0.25% were found in a cheese containing 30% HH milk. Therefore, based on the results obtained so far in this study, both estimated reference values could be used to select suitable PMPs for the α-LA and β-LG in cheese. Taking the SD into account, the β-LG-specific peptides numbered 4, 7, 9, 11, and 12 could be suitable; for α-LA, the peptides numbered 16a, 16b, 18, and 22 seem promising.

**Figure 5:**
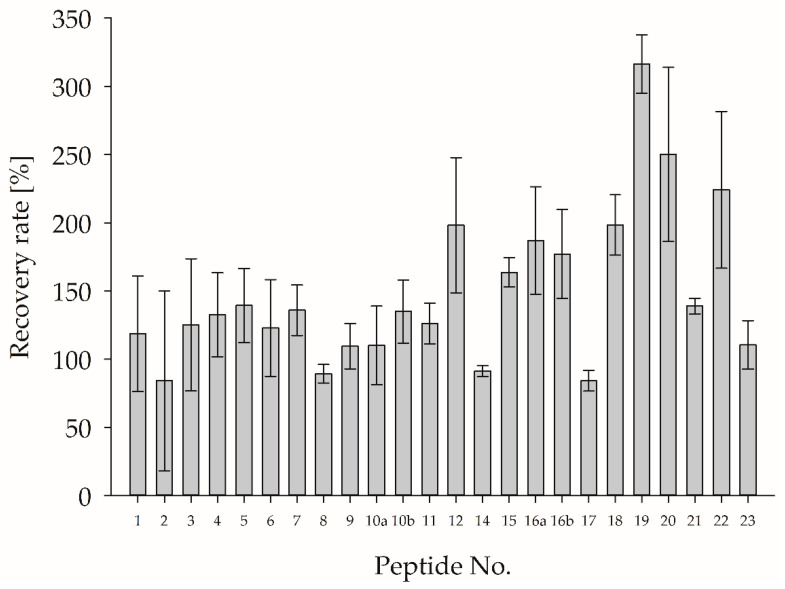
Mean ± SD for the recovery rate of PMP resulted from MPSM spiked to cheese phosphate buffer suspension (CPBS) at three spiking levels prior to tryptic in-vitro hydrolysis. For corresponding peptide sequences, refer to Table 3.

Finally, the recovery rate of MPSM-spiked cheese samples was determined to evaluate whether the degree of hydrolysis of the PMPs out of proteins from the cheese was affected by the cheese matrix during tryptic in-vitro digestion (c.f., Section 2.2.11). Figure 5 shows the calculated recovery rates.

Taking the high SD into account, the recovery rate for most of the identified PMPs for the determination of the β-LG content (peptide no. 1–15) was approximately 100%. The identified PMPs for the determination of the α-LA content (peptide no. 16a–23) showed recovery rates above or below 100%, also with a high SD. In particular, the hydrophobic acidic peptides (no. 12, 19, 20, 22, and 23) showed significantly increased recovery rates, indicating low stability and high matrix effects. To overcome these matrix effects, especially for promising PMP, it is recommended that isotopically labeled internal standards are added prior to tryptic in-vitro hydrolyses. Apparently, a verification of the release of the marker peptides within the product matrix seemed to be necessary for reliable quantification.

Further studies are required to verify suitable marker peptides and subsequently enable reliable quantification of whey proteins occurring in various complex cheese matrices. For further method development, it should be considered that some whey-protein-specific marker peptides are specific for bovine milk or can be applied for identifying whey proteins from various species. In addition, the genetic variants of proteins, such as β-LG A and β-LG B, are relevant, e.g., a tryptic peptide of β-LG B (WENGECAQK) shows a slightly different amino acids sequence in β-LG A (WENDECAQK). Finally, experiments regarding digestibility and stability during tryptic digestion in the different product matrices should be performed, as reported by Hao et al. [29]. The PMPs need to be readily released from their protein and need to be chemically stable during tryptic in-vitro hydrolysis.

## 4. Conclusions

Based on a shotgun-proteomic strategy (‘bottom-up’), PMPs were used to quantify the α-LA and β-LG contents in ripened Edam-type cheese from a pilot plant and an industrial-scale production at a German dairy company. An MRM-LC-MS/MS method was successfully developed. Both the repeatability for multiple tryptic in-vitro hydrolyses and for multiple MRM-LC-MS/MS analyses showed acceptable CVs for most of the PMPs. Furthermore, the analysis of the synthetic PMPs showed reasonable coefficients of determination (R^2^) for the linearity and recovery rates of spiked CPBS and MPSM hydrolysates of approximately 100%. It was shown that α-LA and β-LG were not proteolytically degraded during the cheese-ripening time. Moreover, it was found that the ripening time did not affect the tryptic in-vitro hydrolysis. Based on the analyses of the PMPs, an enrichment of α-LA and β-LG in cheese containing HH milk was confirmed. Therefore, the proposed method could be used to determine the enrichment of α-LA and β-LG in whey-protein-enriched cheese compared to standard cheese (‘relative quantification’). However, absolute quantification of α-LA and β-LG in cheeses from two sample sets, using external calibration, showed low accuracy. As the degree of hydrolysis of the PMPs in MPSMshowed considerable variation, internal calibration was applied to minimize matrix effects and to guarantee that the PMPs from the cheese and the MPSM were hydrolyzed in the same ratio. Thus, better accuracy was achieved but the results still differed for some PMPs. However, the recovery rate of spiked MPSM to CPBS revealed that matrix effects during the tryptic in-vitro digestion are still present. For future studies on the quantification of α-LA and β-LG in cheese, isotopically labeled internal standards are thus recommended to overcome these effects and subsequently select suitable PMPs for valid determination in various product matrices. A fully validated method could be used in perspective for the analysis of the α-LA and β-LG content in ripened cheese to ensure legally compliant products for the German market. Thus, both the quality assurance departments of dairy companies and the official food control authorities can benefit from this analytical method development in order to extend the analytical quality assessment of ripened cheese.

## Figures and Tables

**Table 1:** Overview of analyzed cheese samples; sample set I and sample set II; HH: high-heat milk, BB: before brining, AB: after brining.

Sample Set	HH Milk in Cheese Milk [%] (*w*/*w*)	Replication of Production (n)	Production Stages	
			Cheese Curd before Ripening	Cheese during Ripening [Weeks]
I	0	9	BB, AB	1, 2, 3, 4, 5, 6
	10	3	–	6
	20	3	–	6
	30	3	BB, AB	1, 2, 3, 4, 5, 6
II	0	1	BB, AB	3, 6
	10	1	–	6
	20	1	–	6
	30	1	BB, AB	3, 6

**Table 2:** Individual protein concentration in the milk protein standard mixture.

Protein	Concentration in MPSM [mg mL^−1^]
α-LA	1.08
β-LG (A + B)	2.89
BSA	0.32
LF	0.07
IgG	0.42
α_s_-CN	16.97
β-CN	9.95
κ-CN	2.86

**Table 3:** Data of identified potential marker peptides (PMPs) for quantification of β-LG and α-LA via MRM-LC-MS/MS ^1^.

Protein	No.	Peptide Sequence of PMP	R_t_. [min]	Prec. Mass *m*/*z*	Prec. Charge *z*	Frag. 1 *m*/*z*	Frag. 2 *m*/*z*	Frag. 3 *m*/*z*
β-LG	1	ALPMHIR	13.3	418.9	2	327.2	653.2	425.1
	2	IPAVFK	16.0	337.6	2	281.3	561.2	464.2
	3	LIVTQTMK	13.2	467.4	2	707.1	608.1	227.0
	4	GLDIQK	9.7	337.1	2	503.1	170.9	388.1
	5	WENGEcAQK	7.6	560.9	2	806.0	692.2	158.5
	6	IDALNENK	8.9	458.5	2	688.1	504.1	229.0
	7	VLVLDTDYK	18.0	533.1	2	853.1	754.1	641.0
	8	LSFNPTQLEEQcHI	23.1	859.1	2	928.1	1254.1	815.0
	9	KIIAEK	7.0	351.5	2	460.2	573.1	128.9
	10a	TKIPAVFK [M + 2H]^2+^	15.7	452.0	2	674.2	561.1	343.3
	10b	TKIPAVFK [M + 3H]^3+^	15.7	301.7	3	393.1	256.1	511.0
	11	VLVLDTDYKK	15.3	597.0	2	981.1	882.0	491.2
	12	VYVEELKPTPEGDLEILLQK	26.7	772.3	3	1026.6	791.1	627.9
	13	PMHIR	7.8	327.0	2	425.3	229.1	556.3
	14	TPEVDDEALEKFDK	17.5	546.2	3	512.4	655.0	719.7
	15	TPEVDDEALEK	11.6	623.1	2	819.3	573.1	328.2
α-LA	16a	VGINYWLAHK [M + 3H]^3+^	21.2	400.7	3	551.3	522.6	466.3
	16b	VGINYWLAHK [M + 2H]^2+^	21.2	600.5	2	931.2	654.1	817.1
	17	cEVFR	9.6	355.5	2	421.1	322.0	290.0
	18	IWcK	8.6	303.9	2	493.0	307.1	460.9
	19	DDQNPHSSNIcNIScDK	9.6	668.5	3	736.4	896.5	766.2
	20	FLDDDLTDDIMcVK	24.8	850.3	2	981.3	1439.7	1324.5
	21	LDQWLcEKL	22.5	602.8	2	976.4	848.4	537.2
	22	FLDDDLTDDIMcVKK	23.4	610.0	3	784.3	841.0	555.4
	23	ALcSEKLDQWLcEKL	24.9	631.7	3	588.0	774.8	855.1

^1^ Amino acids are abbreviated according to the IUPAC-IUB Joint Commission on Biochemical Nomenclature (JCBN) standard. c, carbamidomethylated Cys. Abbreviations: R_t_., retention time; Prec., precursor; Frag., fragment.

**Table 4:** Coefficient of variation (CV) for the evaluation of repeatability.

Peptide No.	Potential Marker Peptide	CV Repeatability of Multiple Tryptic Hydrolyses [%]		CV Repeatability of Multiple Measurements [%]		
		Cheese ^a^	MPSM ^b^	Cheese ^c^	MPSM ^c^	Pepmix ^d^
1	ALPMHIR	1.97	4.49	0.43	1.09	1.24
2	IPAVFK	0.78	5.12	0.20	1.15	1.02
3	LIVTQTMK	1.48	6.67	0.53	1.07	1.07
4	GLDIQK	2.18	5.68	0.47	1.61	1.32
5	WENGEcAQK	2.01	8.58	1.34	2.36	2.11
6	IDALNENK	1.70	5.70	0.40	1.62	2.07
7	VLVLDTDYK	1.59	3.91	0.62	0.94	0.92
8	LSFNPTQLEEQcHI	3.17	5.94	1.82	0.98	6.53
9	KIIAEK	3.57	2.22	1.02	1.31	1.36
10a	TKIPAVFK [M + 2H]^2+^	2.67	5.77	0.57	1.07	1.70
10b	TKIPAVFK [M + 3H]^3+^	2.92	6.46	0.89	0.96	1.31
11	VLVLDTDYKK	2.54	2.99	1.20	1.50	2.58
12	VYVEELKPTPEGDLEILLQK	1.85	5.68	0.41	1.03	8.65
13	PMHIR	17.45	48.19	17.76	10.41	1.90
14	TPEVDDEALEKFDK	3.40	1.62	0.43	1.03	1.71
15	TPEVDDEALEK	2.41	2.85	0.51	0.87	1.34
16a	VGINYWLAHK [M + 3H]^3+^	13.68	3.55	0.75	1.06	1.06
16b	VGINYWLAHK [M + 2H]^2+^	11.08	4.53	3.28	0.80	1.77
17	cEVFR	1.94	3.16	0.80	0.92	1.75
18	IWcK	2.61	5.53	0.69	1.36	1.30
19	DDQNPHSSNIcNIScDK	3.98	8.94	1.38	2.54	3.29
20	FLDDDLTDDIMcVK	2.77	7.75	0.84	0.92	3.85
21	LDQWLcEKL	3.51	4.80	0.95	1.12	1.66
22	FLDDDLTDDIMcVKK	7.25	5.69	1.21	1.37	6.56
23	ALcSEKLDQWLcEKL	12.15	5.37	2.82	1.18	9.54

^a^ 8-fold tryptic hydrolysis of one cheese sample, each measured once; ^b^ 5-fold tryptic hydrolysis, each measured once; ^c^ One tryptic hydrolysis, measured ten times; ^d^ Ten injections in a row.

**Table 5:** Mean ± SD for the recovery rate (RC) of synthetic potential marker peptides in different hydrolysates. Mean and SD were calculated from spiking levels 1–3. Sample preparation was performed in duplicate and measured once.

Peptide No.	Potential Marker Peptide	RC_Peptide Spiked CPBS Hydrolysates_ [%]	RC_Peptide Spiked MPSM Hydrolysates_ [%]
1	ALPMHIR	99.8 ± 6.5	106.6 ± 10.1
2	IPAVFK	103.1 ± 1.4	114.5 ± 0.8
3	LIVTQTMK	79.3 ± 1.3	78.1 ± 5.0
4	GLDIQK	99.4 ± 3.5	119.6 ± 3.4
5	WENGEcAQK	94.5 ± 2.1	92.5 ± 1.2
6	IDALNENK	97.6 ± 2.5	107.1 ± 2.4
7	VLVLDTDYK	105.2 ± 1.5	110.9 ± 3.2
8	LSFNPTQLEEQcHI	131.4 ± 22.1	117.2 ± 19.7
9	KIIAEK	97.1 ± 6.1	105.2 ± 3.6
10a	TKIPAVFK [M + 2H]^2+^	95.0 ± 2.0	104.6 ± 3.1
10b	TKIPAVFK [M + 3H]^3+^	92.3 ± 5.2	115.9 ± 1.9
11	VLVLDTDYKK	94.7 ± 3.5	92.5 ± 9.1
12	VYVEELKPTPEGDLEILLQK	n.d.	n.d.
14	TPEVDDEALEKFDK	145.5 ± 5.4	169.1 ± 7.2
15	TPEVDDEALEK	102.0 ± 2.2	112.3 ± 1.9
16a	VGINYWLAHK [M + 3H]^3+^	127.6 ± 10.0	141.9 ± 10.0
16b	VGINYWLAHK [M + 2H]^2+^	125.2 ± 13.2	117.3 ± 12.2
17	cEVFR	70.8 ± 3.5	89.5 ± 5.3
18	IWcK	97.6 ± 3.0	104.7 ± 2.3
19	DDQNPHSSNIcNIScDK	48.0 ± 2.5	53.9 ± 4.9
20	FLDDDLTDDIMcVK	9.5 ± 2.0	3.9 ± 2.5
21	LDQWLcEKL	116.0 ± 14.0	150.2 ± 12.1
22	FLDDDLTDDIMcVKK	n.d.	n.d.
23	ALcSEKLDQWLcEKL	n.d.	n.d.

n.d.: not determined.

**Table 6:** Enrichment factors (EFs) of β-LG for whey protein-enriched cheese from sample set I (pilot plant scale) and sample set II (industrial scale); HH milk: high-heat milk.

Cheese	Sample Set	EF of β-LG Based on Potential Marker Peptides ^a^	EF of β-LG Based on Estimated β-LG Content ^b^
Cheese containing 10% HH milk	I	2.3 ± 0.2	1.9
	II	2.0 ± 0.2	1.9
Cheese containing 20% HH milk	I	3.5 ± 0.4	3.0
	II	3.1 ± 0.4	2.8
Cheese containing 30% HH milk	I	4.8 ± 0.7	4.0
	II	4.1 ± 0.6	3.7

^a^ SD was calculated based on the number of β-LG-specific marker peptides; ^b^ Calculation based on the estimated total individual whey protein content of β-LG. Therefore, the acid-soluble whey protein data resulting from HPLC-UV analyses were used, and the assumption that the ratio of nonacid-soluble protein to denatured whey protein, as present in cheese milk, is also valid for cheese, was made (for detailed description c.f., von Oesen et al. [13]).

**Table 7:** Enrichment factors (EFs) of α-LA for whey protein-enriched cheese from sample set I (pilot plant scale) and sample set II (industrial scale); HH milk: high-heat milk.

Cheese	Sample Set	EF of α-LA Based on Potential Marker Peptides ^a^	EF α-LA Based on Estimated α-LA Content ^b^
Cheese containing 10% HH milk	I	1.8 ± 0.3	1.5
	II	1.6 ± 0.1	1.4
Cheese containing 20% HH milk	I	2.5 ± 0.6	2.6
	II	2.1 ± 0.1	1.9
Cheese containing 30% HH milk	I	3.3 ± 0.9	2.7
	II	2.7 ± 0.2	2.3

^a^ SD was calculated based on the number of α-LA-specific marker peptides; ^b^ Calculation based on the estimated total individual whey protein content of α-LA. Therefore, the acid-soluble whey protein data resulting from HPLC-UV analyses were used, and the assumption that the ratio of nonacid-soluble protein to denatured whey protein, as present in cheese milk, is also valid for cheese, was made (for detailed description c.f., von Oesen et al. [13]).

## Data Availability

The data presented in this study are available on request from the corresponding author. The data are not publicly available due to a very large data set.

## Supplementary Materials

The following supporting information can be downloaded at: https://www.mdpi.com/article/10.3390/foods12102002/s1, Figure S1: peak areas of tryptic in-vitro generated PMPs for α-LA or β-LG throughout the cheese ripening process of sample set I; Figure S2: peak areas of tryptic in-vitro generated PMPs for α-LA or β-LG throughout the cheese ripening process of sample set II; Figure S3: peak areas of PMPs as a function of the amount of HH milk in cheese milk for sample set I and sample set II; Table S1: overview of the volumes used for peptide spiking in duplicate and measured once; Table S2: preparation of the synthetic peptide standard mixture (according to the peptide spiking levels mentioned in Table S1) in duplicate and measured once; Table S3: spiking of CPBS with MPSM prior to tryptic hydrolysis; Table S4: Overview of the sample volumes used for the tryptic hydrolyses of the spiking experiments (Table S3).
