# Supplementary material for: Identification of Marker Peptides for the Whey Protein Quantification in Edam-Type Cheese
Source: Foods. 2023 May 15;12(10):2002. doi: 10.3390/foods12102002 (PMC10217457; doi:10.3390/foods12102002)
Supplement: Supplementary file 1 [file foods-12-02002-s001.zip › foods-2372079-supplementary.pdf]

Supplementary Material

Identification of Marker Peptides for the Whey Protein Quantification in Edam-Type Cheese

Tobias von Oesen, Mascha Treblin, Ingrid Clawin-Rädecker, Dierk Martin, Ronald Maul, Wolfgang Hoffmann, Katrin Schrader, Benjamin Wegner, Katja Bode, Ralf Zink, Sascha Rohn, and Jan Fritsche

Contents

Table S1	Overview of the volumes used for peptide spiking in duplicate and measured once.
Table S2	Preparation of the synthetic peptide standard mixture (according to the peptide spiking levels mentioned in Table S1) in duplicate and measured once.
Table S3	Spiking of CPBS with MPSM prior to tryptic hydrolysis.
Table S4	Overview of the sample volumes used for the tryptic hydrolyses of the spiking experiments (Table S3).
Figure S1	Peak areas of tryptic *in-vitro* generated PMPs for α-LA or β-LG throughout the cheese ripening process of sample set I.
Figure S2	Peak areas of tryptic *in-vitro* generated PMPs for α-LA or β-LG throughout the cheese ripening process of sample set II.
Figure S3	Peak areas of PMPs as a function of the amount of HH milk in cheese milk for sample set I and sample set II.

Table S1. Overview of the volumes used for peptide spiking in duplicate and measured once.

Sample	Hydrolysate [µL]	Added synthetic peptide standard mixture [µL]	Water [µL]
Hydrolysates of CPBS and MPSM	300	0	300
Peptide spiking level 1	300	150	150
Peptide spiking level 2	300	225	75
Peptide spiking level 3	300	300	0

Table S2. Preparation of the synthetic peptide standard mixture (according to the peptide spiking levels mentioned in Table S1) in duplicate and measured once.

Peptide spiking level	Synthetic peptide standard mixture [µL]	Water [µL]
1	150	450
2	225	375
3	300	300

Table S3. Spiking of CPBS with MPSM.

Spiking level	CPBS [µL]	MPSM [µL]	Water [µL]
1	50	50	100
2	50	100	50
3	50	150	0

Table S4. Overview of the sample volumes used for tryptic hydrolysis.

Sample	Volume used for hydrolysis [µL]	Volume of CPBS present in sample volume of the spiked samples [µL]	Volume of MPSM present in sample volume of the spiked samples [µL]	Water [µL]
CPBS	20	–	–	700
MPSM level 1	20	–	–	700
MPSM level 2	40	–	–	680
MPSM level 3	60	–	–	660
Spiking level 1	40	10	10	680
Spiking level 2	40	10	20	680
Spiking level 3	40	10	30	680

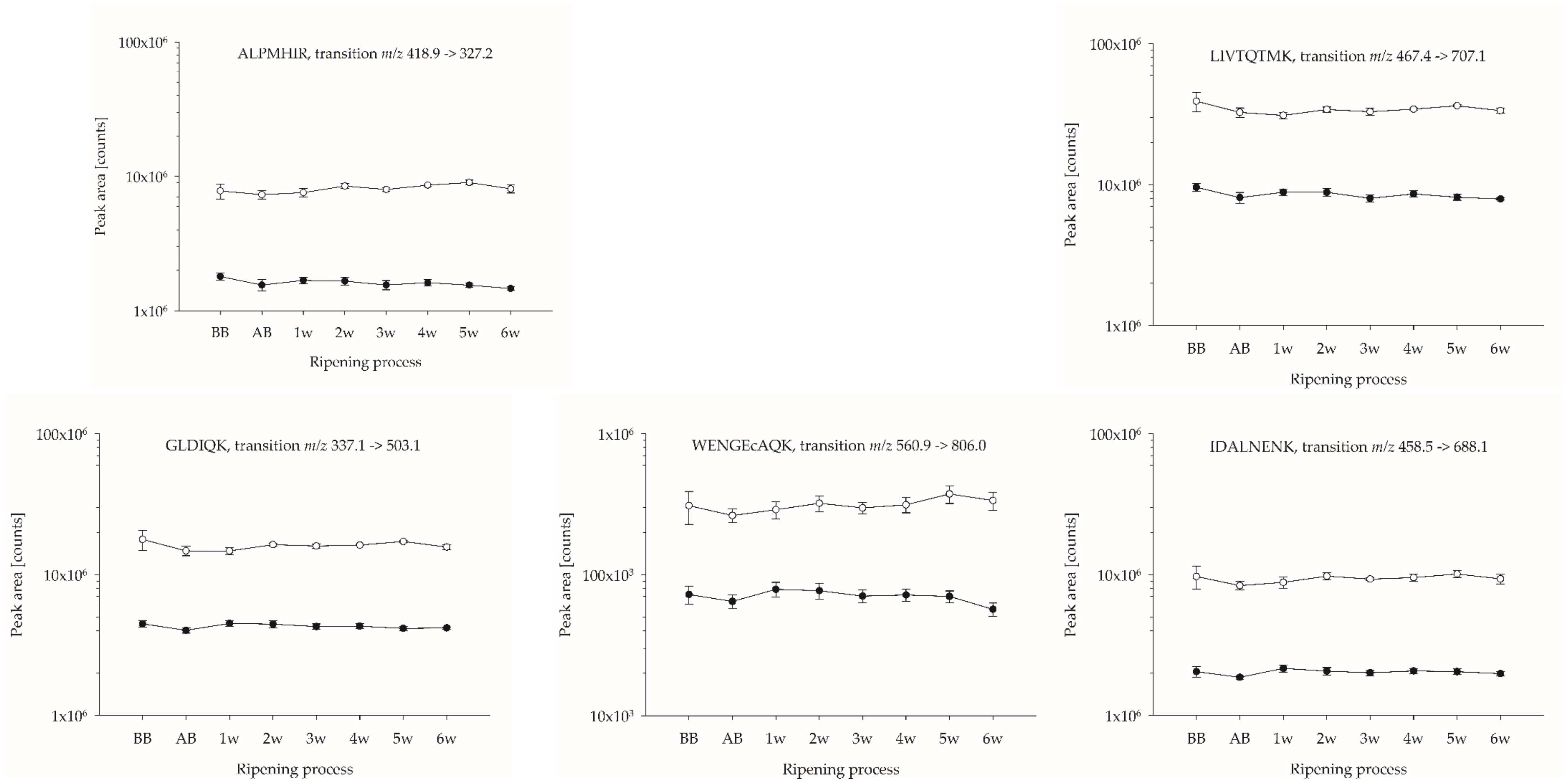
ALPMHIR, transition m/z 418.9 -> 327.2
LIVTQTMK, transition m/z 467.4 -> 707.1
GLDIQK, transition m/z 337.1 -> 503.1
WENGEcAQK, transition m/z 560.9 -> 806.0
IDALNENK, transition m/z 458.5 -> 688.1
Peak area [counts]
100x10^6
10x10^6
1x10^6
100x10^3
10x10^3
BB
AB
1w
2w
3w
4w
5w
6w
Ripening process

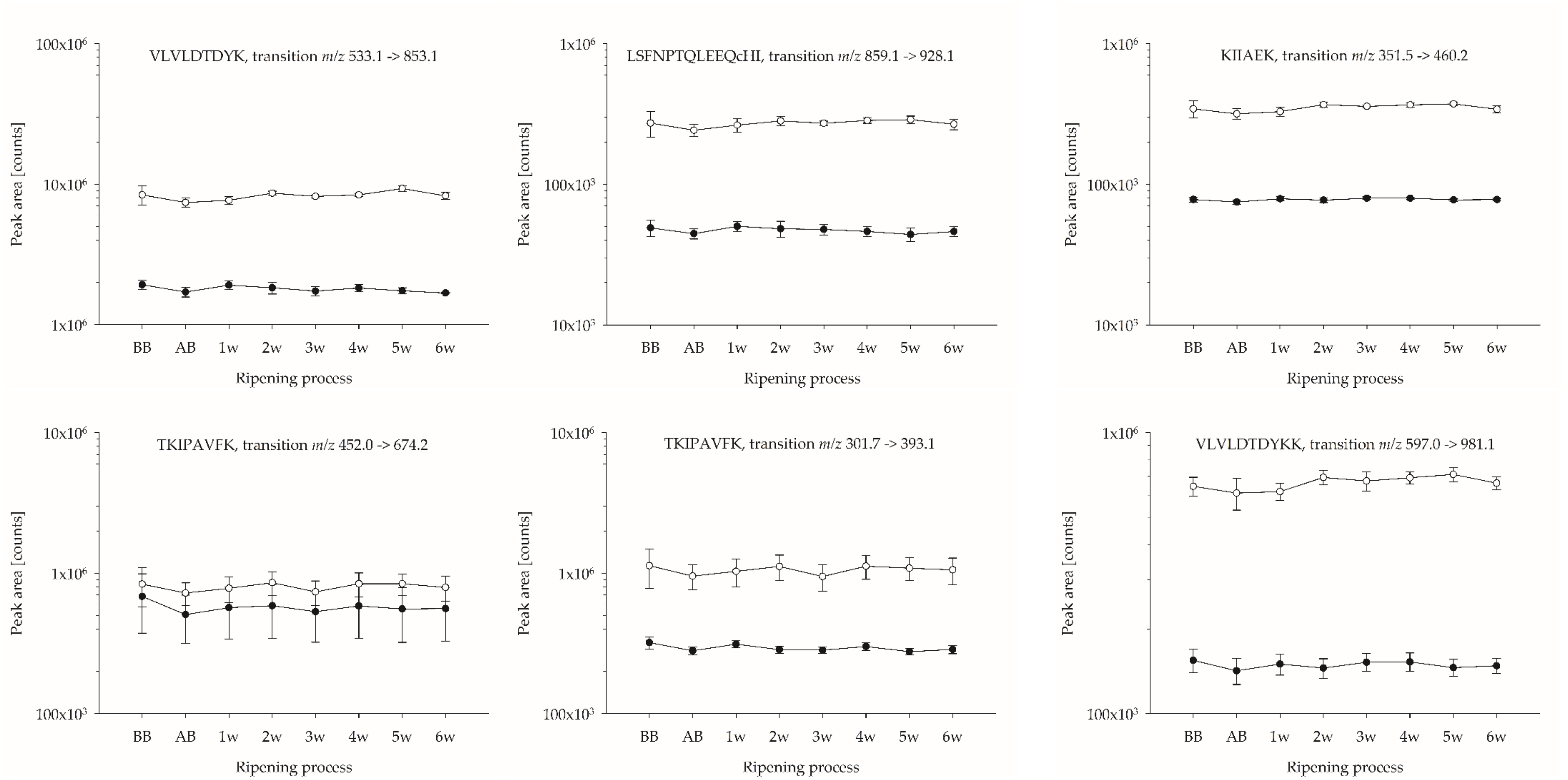

VLVLDTDYK, transition m/z 533.1 -> 853.1
LSFNPTQLEEQcHI, transition m/z 859.1 -> 928.1
KIIAEK, transition m/z 351.5 -> 460.2
TKIPAVFK, transition m/z 452.0 -> 674.2
TKIPAVFK, transition m/z 301.7 -> 393.1
VLVLDTDYKK, transition m/z 597.0 -> 981.1
Peak area [counts]
Ripening process
BB
AB
1w
2w
3w
4w
5w
6w
100x10⁶
10x10⁶
1x10⁶
100x10³
10x10³

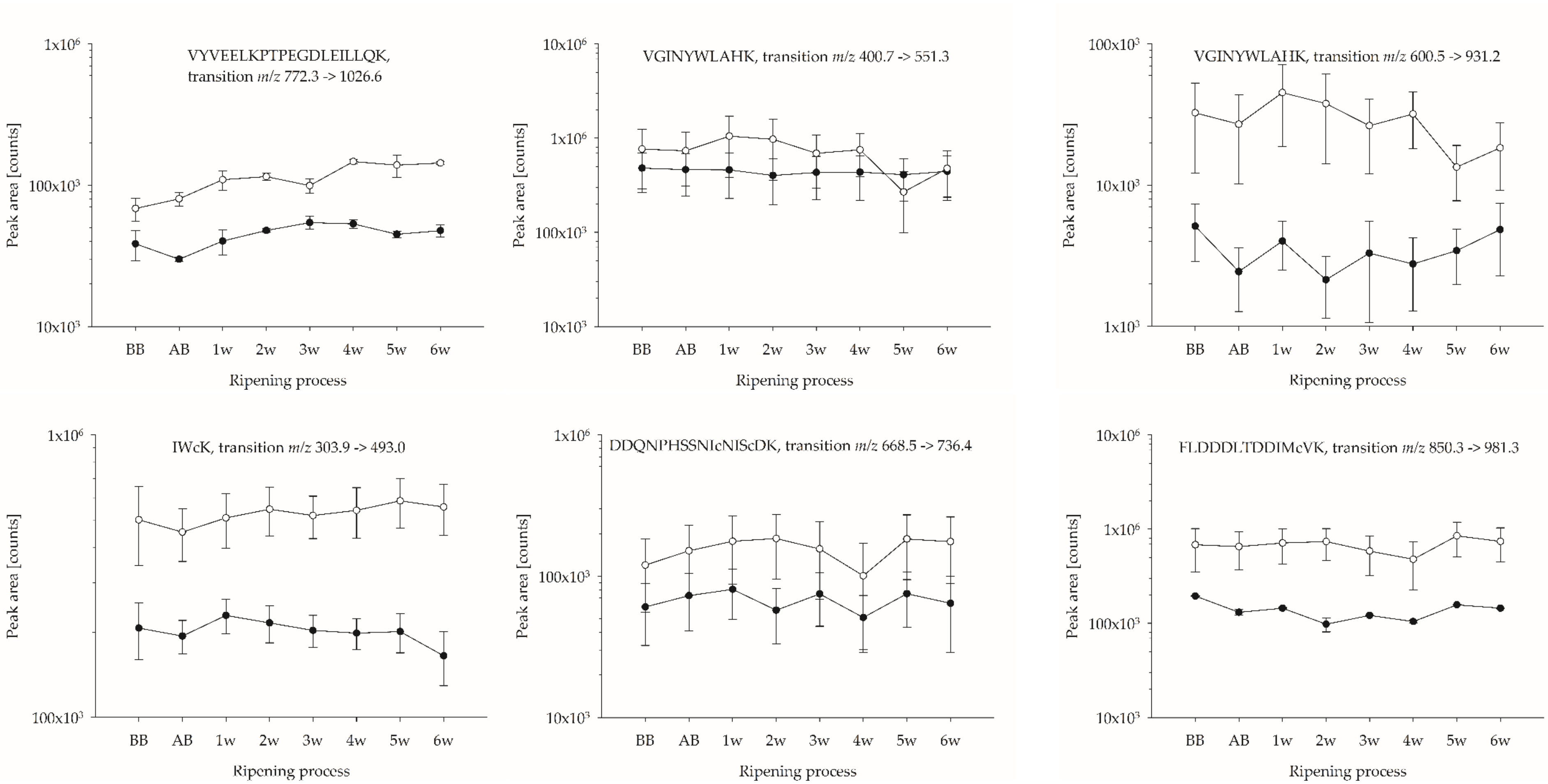
VYVEELKPTPEGDLEILLQK, transition m/z 772.3 -> 1026.6
VGINYWLAHK, transition m/z 400.7 -> 551.3
VGINYWLAHK, transition m/z 600.5 -> 931.2
IWcK, transition m/z 303.9 -> 493.0
DDQNPHSSNIcNIScDK, transition m/z 668.5 -> 736.4
FLDDDLTDDIMcVK, transition m/z 850.3 -> 981.3
Peak area [counts]
Ripening process
BB
AB
1w
2w
3w
4w
5w
6w

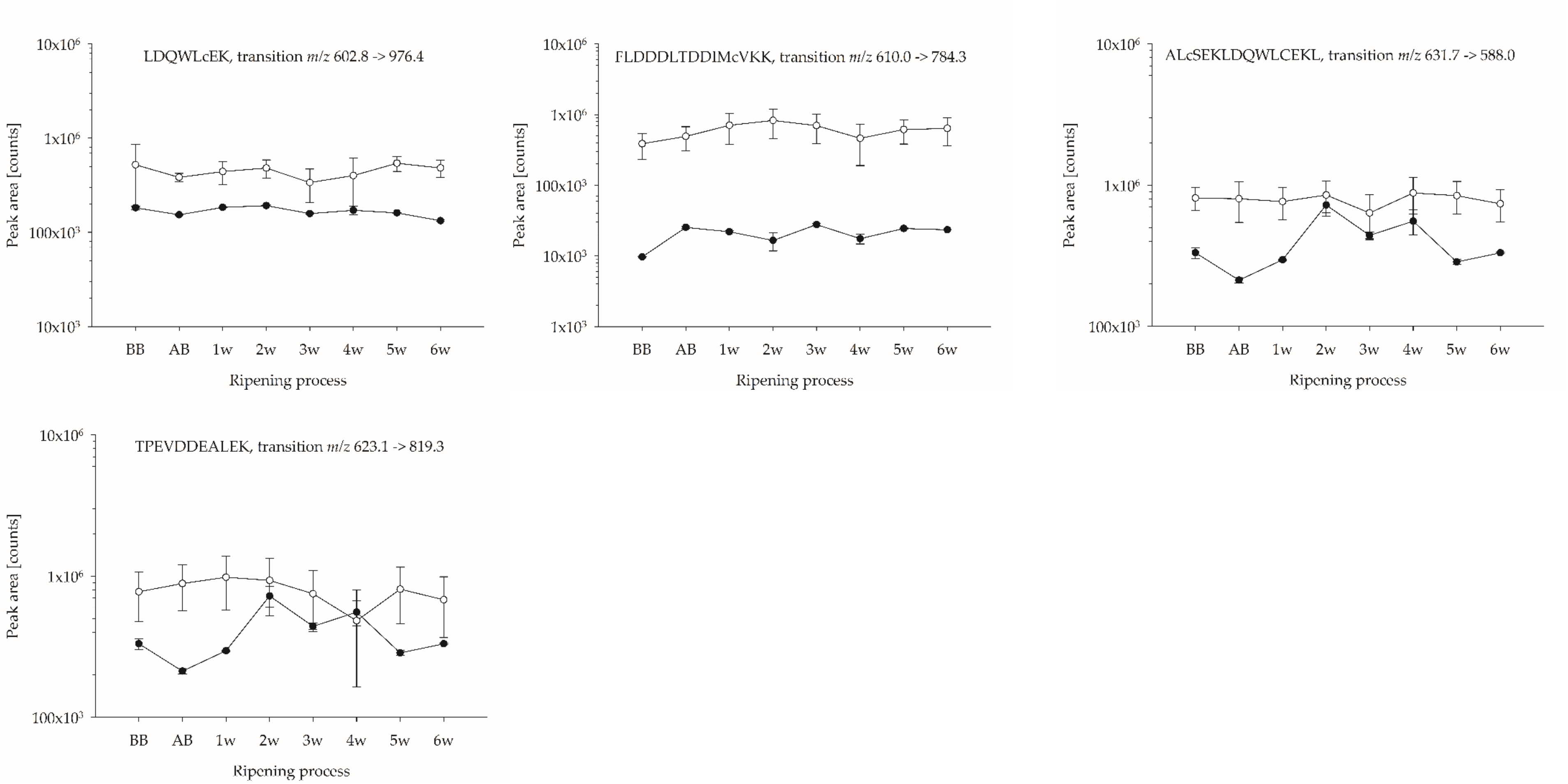

Figure S1. Peak areas of tryptic *in-vitro* generated PMPs for α-LA or β-LG throughout the cheese ripening process of sample set I. For graphical representation, the y-axis was displayed logarithmically. Data were calculated as mean ± SD of three replicate productions; (●) cheese containing 0% HH milk; (○) cheese containing 30% HH milk. Sample preparation was carried out in duplicate and measured once.

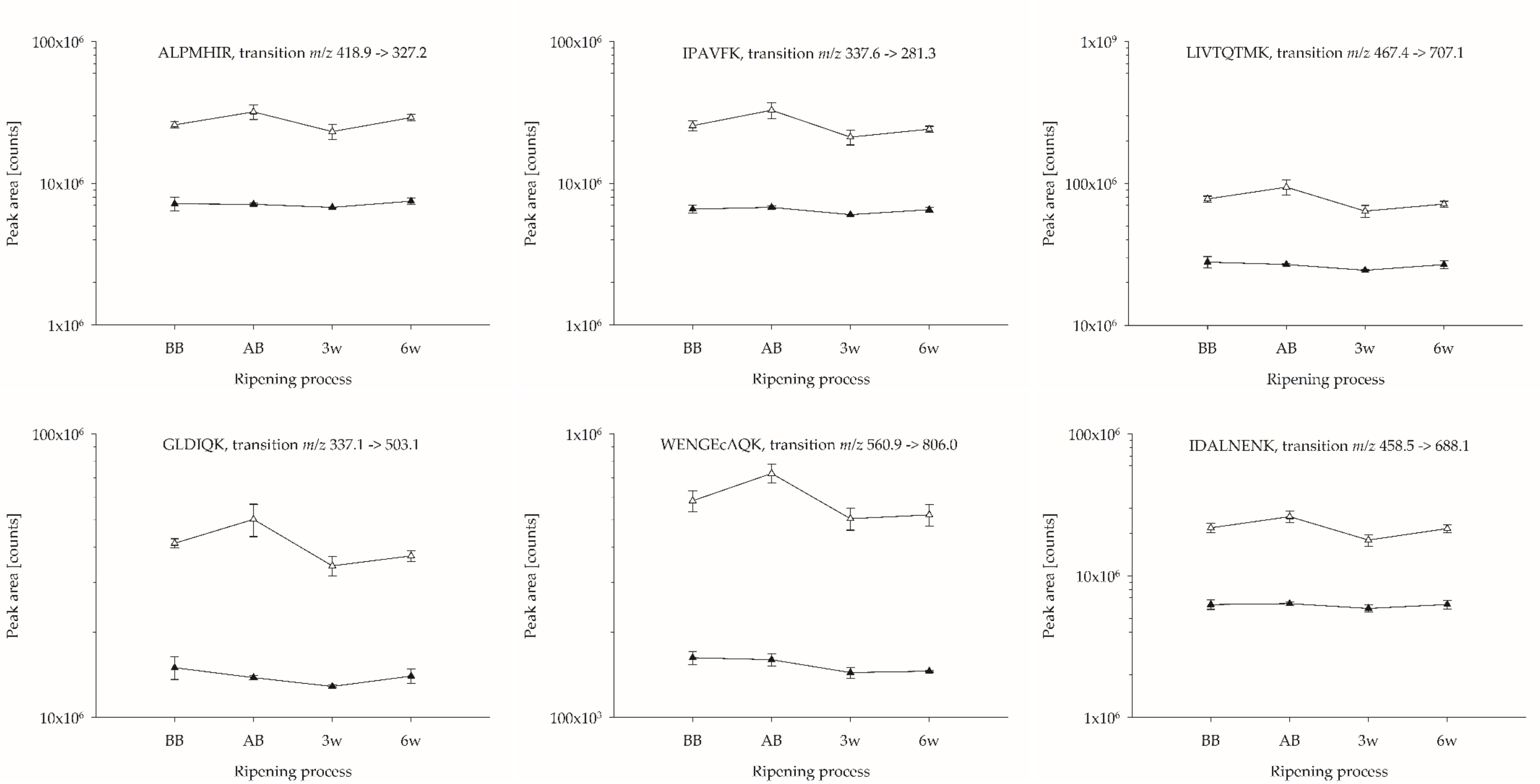

ALPMHIR, transition m/z 418.9 -> 327.2
IPAVFK, transition m/z 337.6 -> 281.3
LIVTQTMK, transition m/z 467.4 -> 707.1
GLDIQK, transition m/z 337.1 -> 503.1
WENGEcAQK, transition m/z 560.9 -> 806.0
IDALNENK, transition m/z 458.5 -> 688.1
Peak area [counts]
Ripening process
BB
AB
3w
6w
100x10^6
10x10^6
1x10^6
1x10^9
100x10^3

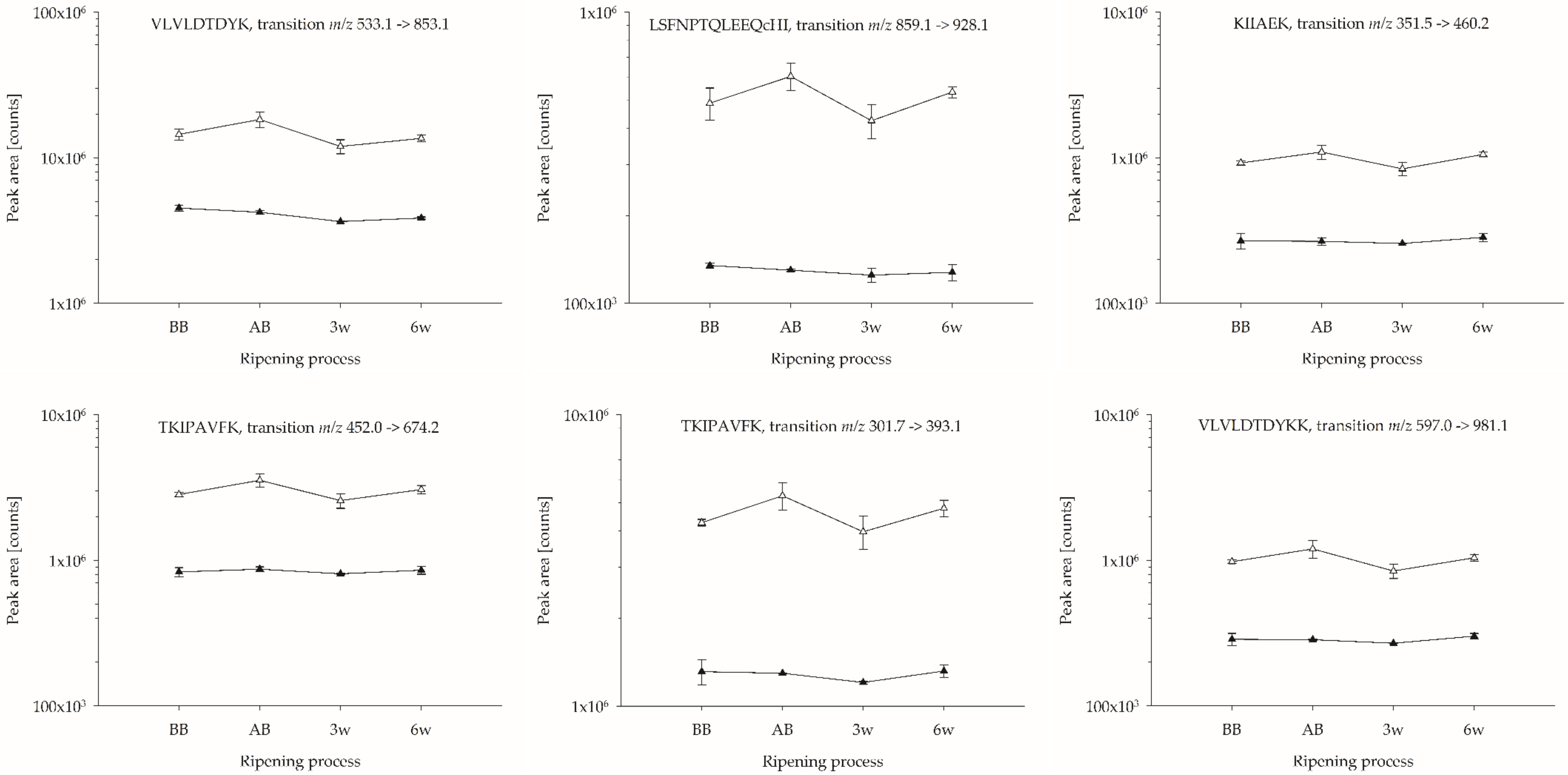
VLVLDTDYK, transition m/z 533.1 -> 853.1
LSFNPTQLEEQcHI, transition m/z 859.1 -> 928.1
KHAEK, transition m/z 351.5 -> 460.2
TKIPAVFK, transition m/z 452.0 -> 674.2
TKIPAVFK, transition m/z 301.7 -> 393.1
VLVLDTDYKK, transition m/z 597.0 -> 981.1
Peak area [counts]
Ripening process
BB
AB
3w
6w
100x10^6
10x10^6
1x10^6
100x10^3

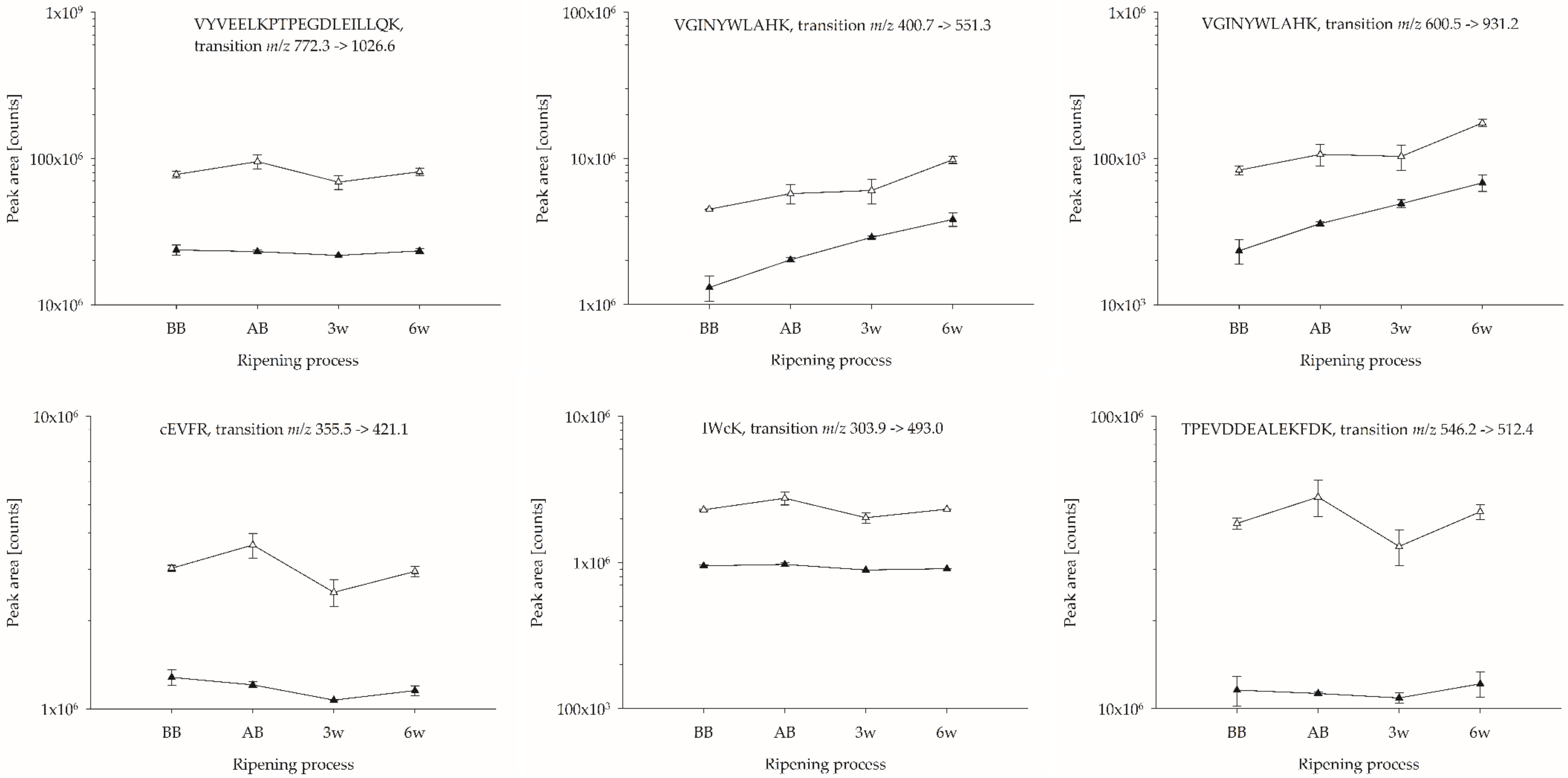

VYVEELKPTPEGDLEILLQK, transition m/z 772.3 -> 1026.6
VGINYWLAHK, transition m/z 400.7 -> 551.3
VGINYWLAHK, transition m/z 600.5 -> 931.2
cEVFR, transition m/z 355.5 -> 421.1
IWcK, transition m/z 303.9 -> 493.0
TPEVDDEALEKFDK, transition m/z 546.2 -> 512.4
Peak area [counts]
Ripening process
BB
AB
3w
6w
1x10^9
100x10^6
10x10^6
1x10^6
1x10^6
100x10^3
10x10^3
100x10^3

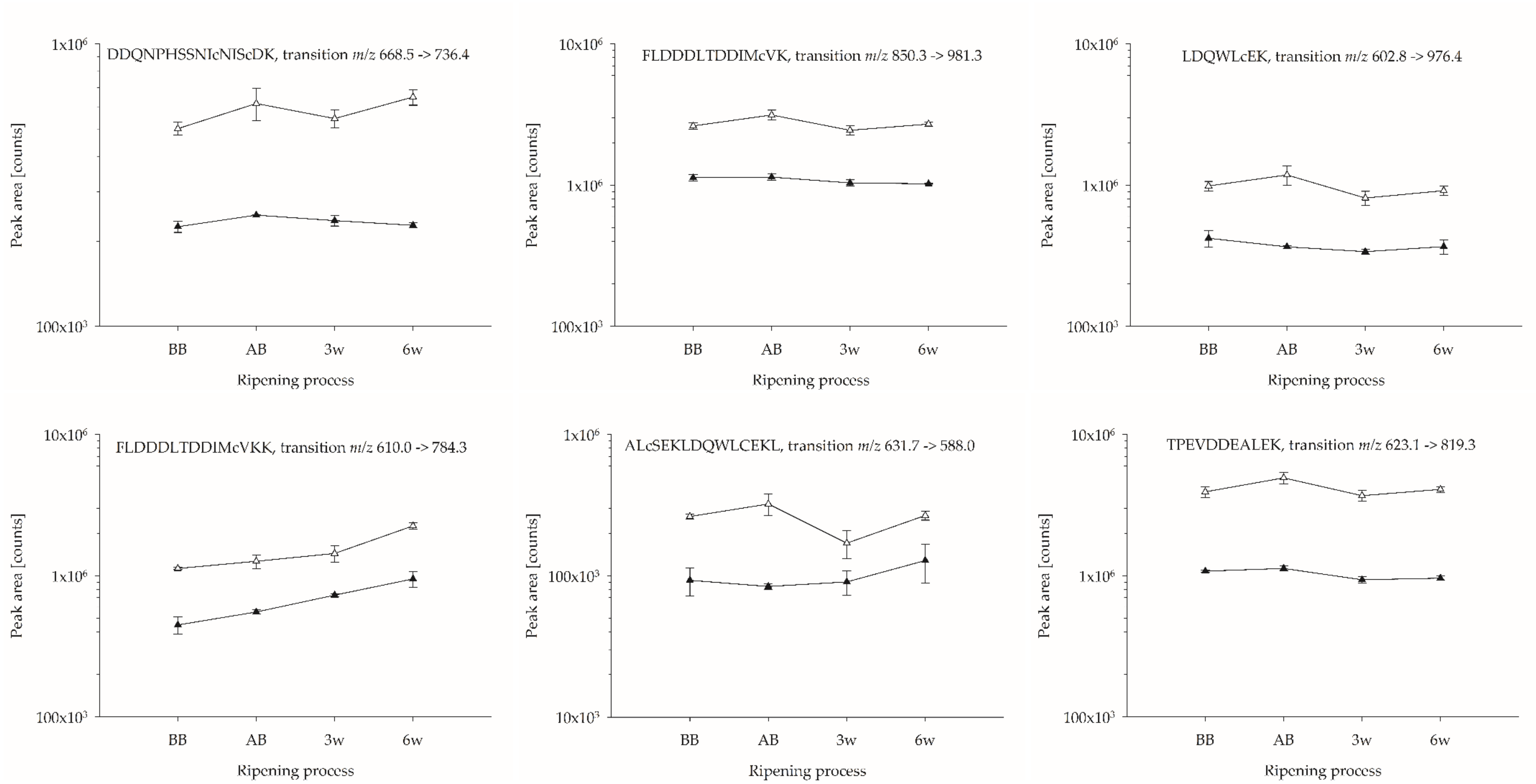

Figure S2. Peak areas of tryptic *in-vitro* generated PMPs for α-LA or β-LG throughout the cheese ripening process of sample set II. For graphical representation, the y-axis was displayed logarithmically. Data were calculated as mean ± SD of a sample preparation in duplicate and measured once; (▲) cheese containing 0% HH milk, (△) cheese containing 30% HH milk.

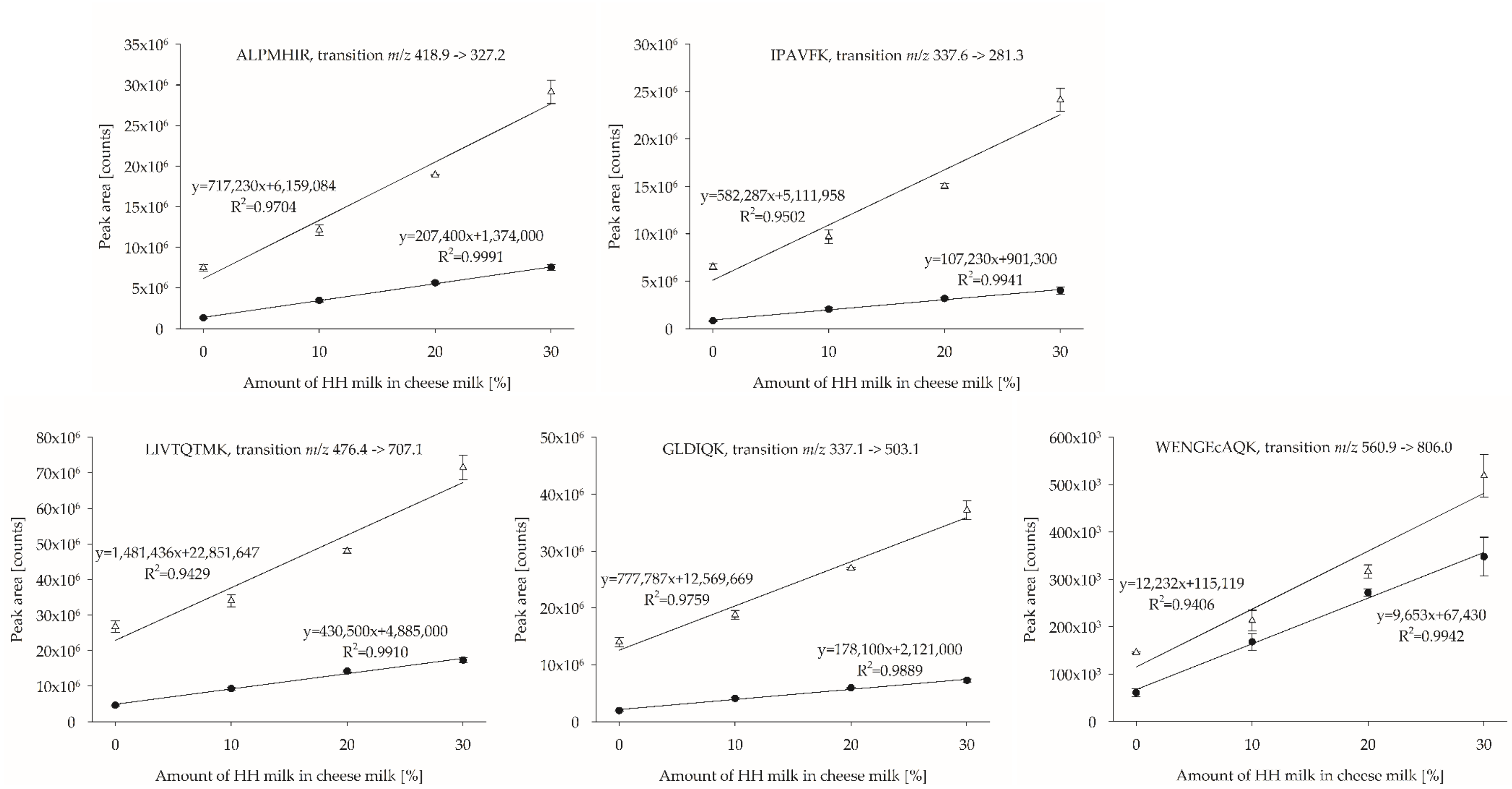

ALPMHIR, transition m/z 418.9 -> 327.2
y=717,230x+6,159,084
R2=0.9704
y=207,400x+1,374,000
R2=0.9991
IPAVFK, transition m/z 337.6 -> 281.3
y=582,287x+5,111,958
R2=0.9502
y=107,230x+901,300
R2=0.9941
LIVTQTMK, transition m/z 476.4 -> 707.1
y=1,481,436x+22,851,647
R2=0.9429
y=430,500x+4,885,000
R2=0.9910
GLDIQK, transition m/z 337.1 -> 503.1
y=777,787x+12,569,669
R2=0.9759
y=178,100x+2,121,000
R2=0.9889
WENGEcAQK, transition m/z 560.9 -> 806.0
y=12,232x+115,119
R2=0.9406
y=9,653x+67,430
R2=0.9942
Peak area [counts]
Amount of HH milk in cheese milk [%]

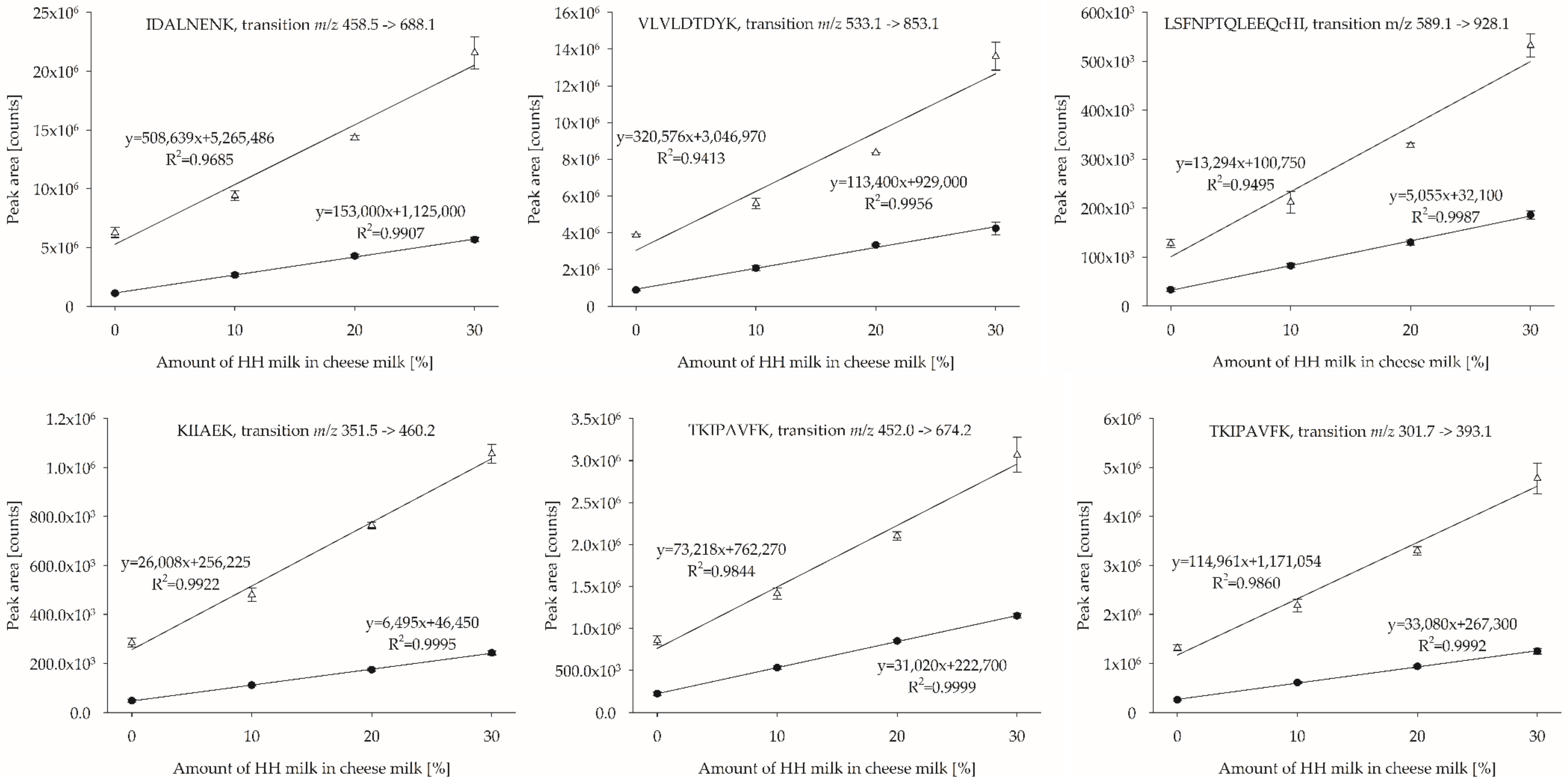

IDALNENK, transition m/z 458.5 -> 688.1
y=508,639x+5,265,486
R2=0.9685
y=153,000x+1,125,000
R2=0.9907
VLVLDTDYK, transition m/z 533.1 -> 853.1
y=320,576x+3,046,970
R2=0.9413
y=113,400x+929,000
R2=0.9956
LSFNPTQLEEQcHI, transition m/z 589.1 -> 928.1
y=13,294x+100,750
R2=0.9495
y=5,055x+32,100
R2=0.9987
KIIAEK, transition m/z 351.5 -> 460.2
y=26,008x+256,225
R2=0.9922
y=6,495x+46,450
R2=0.9995
TKIPAVFK, transition m/z 452.0 -> 674.2
y=73,218x+762,270
R2=0.9844
y=31,020x+222,700
R2=0.9999
TKIPAVFK, transition m/z 301.7 -> 393.1
y=114,961x+1,171,054
R2=0.9860
y=33,080x+267,300
R2=0.9992
Peak area [counts]
Amount of HH milk in cheese milk [%]

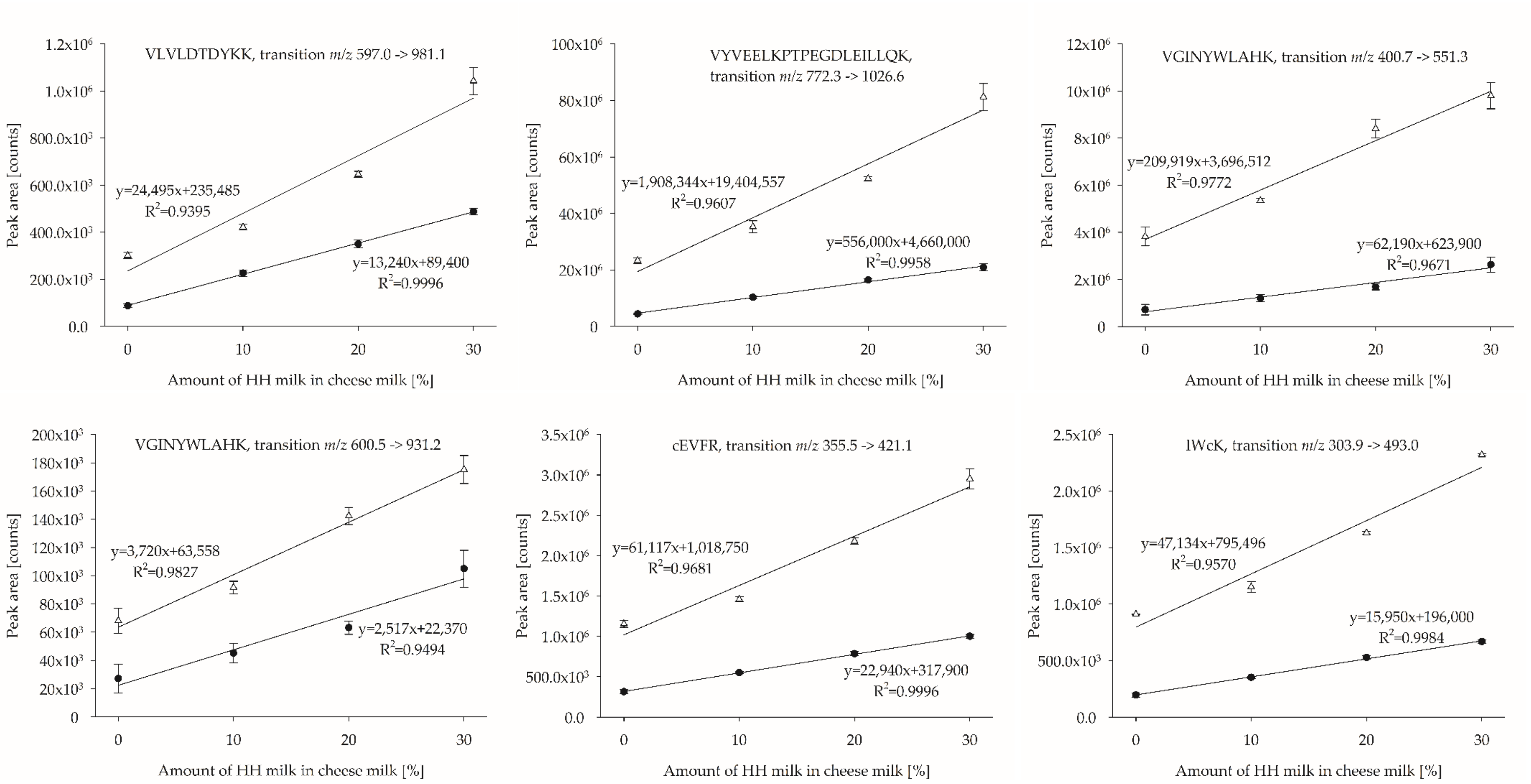
VLVLDTDYKK, transition m/z 597.0 -> 981.1
y=24,495x+235,485
R2=0.9395
y=13,240x+89,400
R2=0.9996
VYVEELKPTPEGDLEILLQK,
transition m/z 772.3 -> 1026.6
y=1,908,344x+19,404,557
R2=0.9607
y=556,000x+4,660,000
R2=0.9958
VGINYWLAHK, transition m/z 400.7 -> 551.3
y=209,919x+3,696,512
R2=0.9772
y=62,190x+623,900
R2=0.9671
VGINYWLAHK, transition m/z 600.5 -> 931.2
y=3,720x+63,558
R2=0.9827
y=2,517x+22,370
R2=0.9494
cEVFR, transition m/z 355.5 -> 421.1
y=61,117x+1,018,750
R2=0.9681
y=22,940x+317,900
R2=0.9996
IWcK, transition m/z 303.9 -> 493.0
y=47,134x+795,496
R2=0.9570
y=15,950x+196,000
R2=0.9984
Peak area [counts]
Amount of HH milk in cheese milk [%]

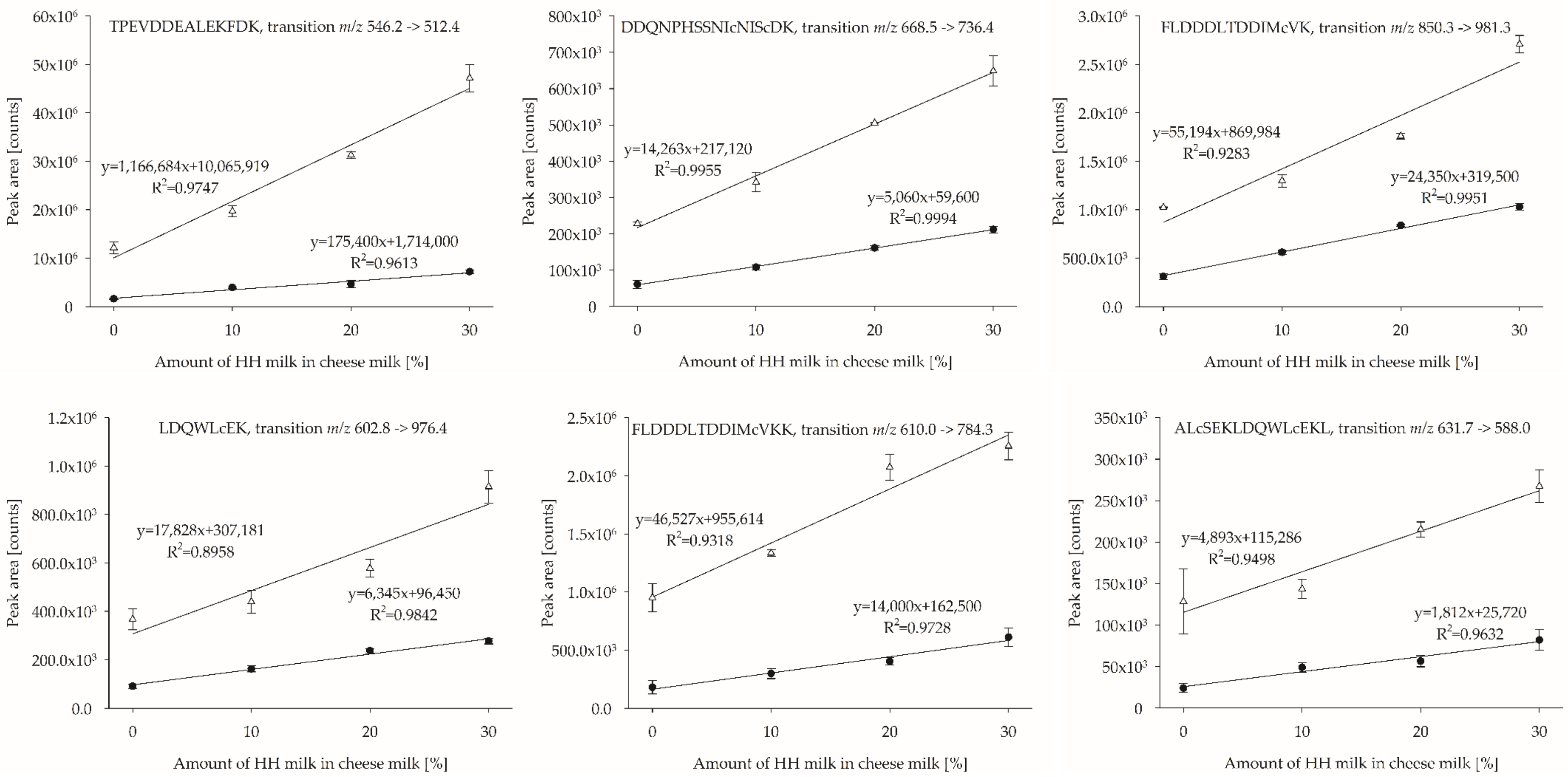
TPEVDDEALEKFDK, transition m/z 546.2 -> 512.4
y=1,166,684x+10,065,919
R2=0.9747
y=175,400x+1,714,000
R2=0.9613
DDQNPHSSNIcNIScDK, transition m/z 668.5 -> 736.4
y=14,263x+217,120
R2=0.9955
y=5,060x+59,600
R2=0.9994
FLDDDLTDDIMcVK, transition m/z 850.3 -> 981.3
y=55,194x+869,984
R2=0.9283
y=24,350x+319,500
R2=0.9951
LDQWLcEK, transition m/z 602.8 -> 976.4
y=17,828x+307,181
R2=0.8958
y=6,345x+96,450
R2=0.9842
FLDDDLTDDIMcVKK, transition m/z 610.0 -> 784.3
y=46,527x+955,614
R2=0.9318
y=14,000x+162,500
R2=0.9728
ALcSEKLDQWLcEKL, transition m/z 631.7 -> 588.0
y=4,893x+115,286
R2=0.9498
y=1,812x+25,720
R2=0.9632
Peak area [counts]
Amount of HH milk in cheese milk [%]

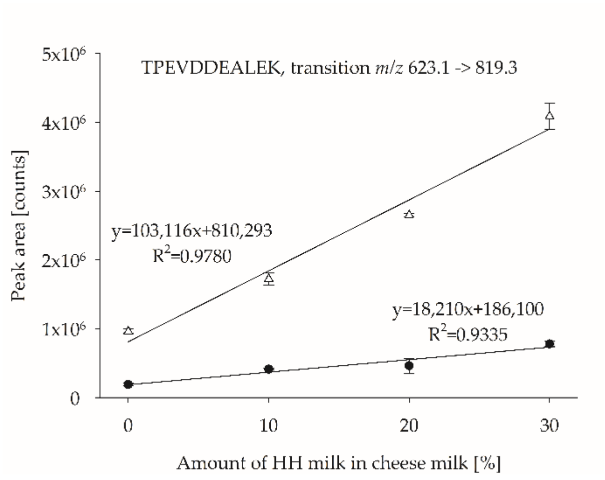

Figure S3. Peak areas of PMPs as a function of the amount of HH milk in cheese milk for sample set I (●) and sample set II (△). R^2 stands for the coefficient of determination. Data points were calculated as mean ± SD. Sample preparation was carried out in duplicate and measured once.
